# Generation of murine tumor models refractory to αPD-1/-L1 therapies due to defects in antigen processing/presentation or IFNγ signaling using CRISPR/Cas9

**DOI:** 10.1371/journal.pone.0287733

**Published:** 2024-03-01

**Authors:** Paul L. Chariou, Christine M. Minnar, Mayank Tandon, Mary R. Guest, Raj Chari, Jeffrey Schlom, Sofia R. Gameiro

**Affiliations:** 1 Center for Immuno-Oncology, Center for Cancer Research, National Cancer Institute, National Institutes of Health, Bethesda, MD, United States of America; 2 National Cancer Institute, CCR Collaborative Bioinformatics Resource, Center for Cancer Research, National Institutes of Health, Bethesda, MD, United States of America; 3 Advanced Biomedical Computational Science, Frederick National Laboratory for Cancer Research, Frederick, MD, United States of America; 4 Laboratory Animal Sciences Program, Frederick National Laboratory for Cancer Research, Frederick, MD, United States of America; Hirosaki University Graduate School of Medicine, JAPAN

## Abstract

Immune checkpoint blockade (ICB) targeting the programmed cell death protein 1 (PD-1) and its ligand 1 (PD-L1) fails to provide clinical benefit for most cancer patients due to primary or acquired resistance. Drivers of ICB resistance include tumor antigen processing/presentation machinery (APM) and IFNγ signaling mutations. Thus, there is an unmet clinical need to develop alternative therapies for these patients. To this end, we have developed a CRISPR/Cas9 approach to generate murine tumor models refractory to PD-1/-L1 inhibition due to APM/IFNγ signaling mutations. Guide RNAs were employed to delete *B2m*, *Jak1*, or *Psmb9* genes in ICB-responsive EMT6 murine tumor cells. B2m was deleted in ICB-responsive MC38 murine colon cancer cells. We report a detailed development and validation workflow including whole exome and Sanger sequencing, western blotting, and flow cytometry to assess target gene deletion. Tumor response to ICB and immune effects of gene deletion were assessed in syngeneic mice. This workflow can help accelerate the discovery and development of alternative therapies and a deeper understanding of the immune consequences of tumor mutations, with potential clinical implications.

## Introduction

State-of-the-art technological achievements in large-scale cloning, genome editing, and DNA/RNA-sequencing have enabled and accelerated breakthrough discoveries in the fields of biology and medicine [1,2]. Notably, CRISPR/Cas9 has become a versatile gene editing tool to study relations between genome and proteome phenotypes, to generate cell and animal models of disease, and to perturb disease progression [3,4]. Compared to earlier generation tools, including transcription activator-like effector nucleases (TALEN) and zinc finger nucleases (ZFN), CRISPR/Cas9 is less time-consuming, cheaper, and user-friendly [4]. Although most of its application has been in basic research, there is an unmet need to capitalize on CRISPR/Cas9 robustness and flexibility to generate cellular tools directly applicable to translational immuno-oncology.

CRISPR/Cas9 can be harnessed to streamline the development of cancer therapies by introducing loss-of-function and gain-of-function mutations in cancer cells. For example, CRISPR screens have been used to identify chemotherapeutic drug targets [5,6]. CRISPR/Cas9-mediated deletion of the viral oncogenes E6 and E7 from the human papillomavirus in cervical cancer cell lines was shown to restore cell cycle arrest and cell death, opening the path to translate CRISPR-mediated E6 and E7 deletion to clinical use [7,8]. Deleting the *PDCD1* gene encoding the immune checkpoint inhibitor programmed cell death protein 1 (PD-1) in T lymphocytes significantly enhanced their immune function [9]. Several ongoing clinical trials are now evaluating adoptive transfer of PD-1-deleted T cells in patients harboring advanced non-small cell lung (NSCLC), bladder, renal cell, prostate, and esophageal cancers [9]. While CRISPR/Cas9 has been applied to the discovery of alternative cancer therapies, there is currently no streamlined approach to generate and validate murine solid cancer models refractory to immune checkpoint blockade (ICB).

Blockade of immune checkpoints such as PD-1 and its ligand PD-L1 has achieved unprecedented success across malignancies, particularly in the combination setting, including in melanoma, renal cell, high microsatellite instable (MSI-H) colorectal, triple-negative breast cancer (TNBC) and bladder cancers, and NSCLC, where tumor PD-L1 expression is observed in 14% to 100% of patients [10]. In one example, pembrolizumab (anti-PD-1) and atezolizumab (anti-PD-L1) are now approved for the treatment of patients with PD-L1^+^ TNBC [11]. However, tumor PD-L1 expression ranging from 7% to 53% is also observed accross ICB-refractory malignancies, such as prostate and micro-satellite stable colorectal cancer, indicating that PD-L1 is not the sole predictor of response [10]. Irrespective of PD-L1 expression, clinical benefit remains hindered by the development of primary or acquired resistance [12]. Other factors including low abundance of tumor-necrosis factor at the tumor site have been shown to correlate with lack of response to ICB [13]. Gene aberrations in antigen processing machinery (APM), including tumor-intrinsic loss of major histocompatibility complex class I (MHC-I), and mutations in the IFNγ signaling pathway are well-documented hallmarks of resistance to PD-1/PD-L1 blockade [14–17] in various cancers and can potentially hinder patient response to other immunotherapies, including adoptive T cell therapies and therapeutic cancer vaccines. There is an unmet critical need to develop alternative treatments for ICB-refractory patients harboring these mutations. While a spectrum of therapies is being examined for the treatment of ICB-resistant patients, there is a lack of preclinical models of solid tumors reflecting the specific gene loss-of-function mutations driving patient resistance to ICB.

Here we seek to directly address this gap through the development of a CRISPR/Cas9 approach to generate and validate translationally relevant tumor models refractory to PD-1/-L1 blockade. Two murine tumor cell lines encompassing a range of positive response to ICB were selected as proof-of-concept models. The TNBC EMT6 model has moderate response to PD-1/-L1 ICB, whereas the colon cancer model MC38 attains significant response [18–20]. As a benchmark, we knocked out the APM genes *B2m* (encoding β_2_ microglobulin) in both cell lines, and *Psmb9* (encoding LMP2) plus the IFNγ signaling pathway gene *Jak1* (encoding Janus kinase 1) in EMT6 cells. Deletion of tumor cell *B2m* results in the loss of MHC class I, thereby preventing tumor recognition and elimination by CD8^+^ T cells [21]. Similarly, deletion of LMP2 prevents the degradation of endogenous tumor antigens in the cytosol, averting their loading onto MHC class I molecules and subsequent presentation to cognate T lymphocytes [22]. Following antigen recognition, IFNγ produced by T cells activates the IFNγ receptor, resulting in the expression of IFNγ-stimulated genes, including PD-L1, through Janus kinase 1 (Jak1) signaling [23]. Jak1 further regulates the expression of chemokines driving tumor T cell infiltration, such as CXCL9, CXCL10 and CXCL11 [24]. Thus, Jak1 deletion directly averts PD-L1 protein expression, devoiding the tumor cell of a primary target of immune checkpoint blockade, and suppresses T cell infiltration in the tumor microenvironment (TME) [23].

CRISPR/Cas9 off-target gene editing is one of the major hurdles of this technology [25]. To address this caveat, whole exome sequencing (WES) was complemented with Sanger sequencing to detect any CRISPR-induced off-target mutations. Gene deletions were further confirmed by western blotting and flow cytometry. The generated models were implanted in syngeneic murine hosts to assess the effects of targeted tumor cell gene deletions on the tumor immunome. Lastly, the resistance of gene knock-out tumor models to αPD-1/αPD-L1 therapeutics was confirmed in syngeneic mice.

## Results

### The production of a successful CRISPR KO clone requires a rigorous funnel down validation

**Fig 1:**
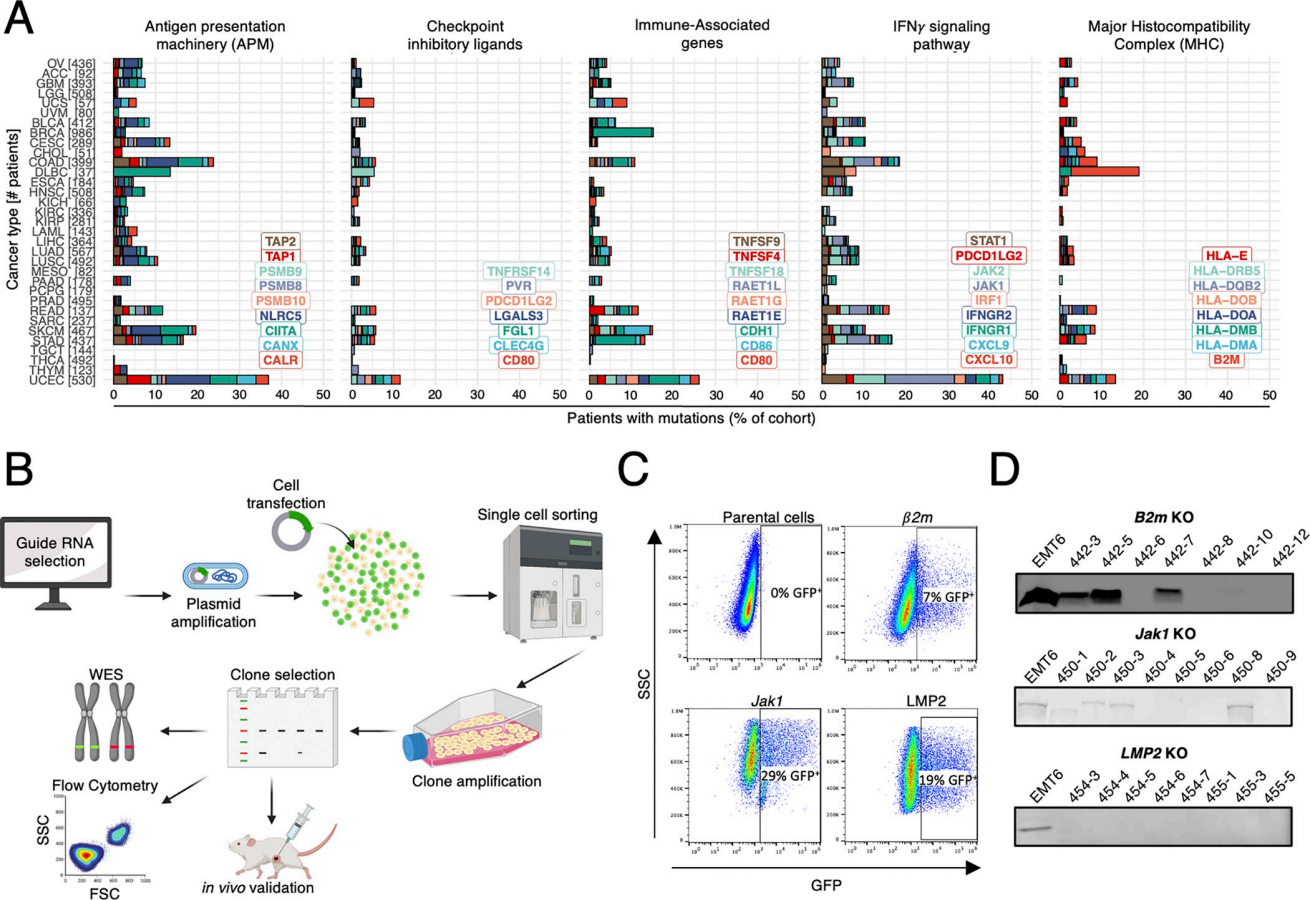
Generation of murine monoclonal tumor models of resistance to immune checkpoint inhibitors using CRISPR/Cas9. (A) Frequency of gene alterations in human cancers across TCGA datasets. Examined gene lists (insets) are subdivided into antigen presentation machinery, checkpoint inhibitory ligands, immune-associated genes, IFNγ signaling pathway, and MHC class I. Genomic alterations include all non-silent single nucleotide polymorphisms (SNPs) and indels. (B) Schematic workflow for the development and validation of CRISPR gene knockout murine cell lines. (C) Representative gating strategy used to isolate transfected (GFP^+^) single cells by flow cytometry. Insets denote percentage of GFP^+^ cells. (D) B2m, Jak1 and LMP2 protein expression in 7–8 individual clones versus wild type EMT6 cells.

Cancer genetic instability frequently leads to loss of expression of cellular components through epigenetic silencing, gene deletion or loss-of-function mutations. Gene aberrations in APM, including loss of 20S proteasome subunits and tumor-intrinsic loss of MHC-I, as well as mutations in IFNγ response pathway genes have been reported as hallmarks of PD-1 or PD-L1 ICB resistance due to loss of tumor immune recognition [14–17]. To quantify the frequency of such mutations, publicly available solid cancer datasets from The Cancer Genome Atlas (TCGA) were retrieved using the TCGABiolinks R package (version 2.20.1) [26]. For clinical relevance, analysis was restricted to non-silent mutations, defined as the 12 variant classifications with “High” or “Moderate” impact reported by the Variant Effect Predictor (VEP) [27]. Single nucleotide polymorphisms (SNPs) and small insertions/deletions (indels) were analyzed across 33 solid cancer types, and curated into five categories: APM (9 genes), MHC (8 genes), checkpoint inhibitor ligands (7 genes), IFNγ pathway (9 genes), and immune-associated markers (9 genes) (Fig 1A). While the frequency of patients displaying each singular mutation is relatively low, mutations in multiple genes in each pathway were observed across all tumor types examined.

To fast-track the discovery of treatment options for patients who fail to respond to PD-1 or PD-L1 ICB, we sought to develop and validate a streamed pipeline to generate well-defined murine solid tumors refractory to these checkpoint inhibitors (Fig 1B). To this end, a CRISPR/Cas9-based method was used to delete single genes encoding proteins whose mutations/deletions are clinically associated with the development of PD-1 or PD-L1 ICB resistance. Multiple candidate guide RNAs targeting the APM genes *B2m* (encoding beta-2 microglobulin), *Psmb9* (encoding the proteasome 20S subunit beta type-9/LMP2), and the IFNγ signaling pathway gene *Jak1* (encoding Janus kinase 1) were identified using the sgRNA Scorer 2.0 algorithm (S1 Table in S1 File). Two guide RNAs per target gene were selected, annealed, and ligated into the commercially available BsmBI-digested Lenti-CRISPRv2GFP (Addgene, Watertown, MA) plasmid (see Methods for details). Compared to other CRISPR/Cas9 approaches, this plasmid contains a green fluorescence protein (GFP) sequence, which allows for the positive selection of successfully transfected cells using fluorescence-activated cell sorting (FACS). GFP expression is lost upon cell replication. This method yielded a transfection efficiency varying between 7% and 29% (Fig 1C). It is worth noting that the transfection efficiency can be optimized. However, that was not required in this setting since only 96 GFP^+^ cells out of the 2 million transfected cells (hence 0.048%) were selected for single-cell clonal expansion. Individually seeded cells were allowed to replicate for 2 weeks in 96-well plates, after which 10 clones were randomly selected for each single-gene knockout (KO) cell line and transferred onto 12-well plates, then onto T-150 culture flasks for further expansion. Following clonal expansion of each gene KO cell line, cell lysates of 7–8 randomly selected EMT6 clones were analyzed by western blot for the protein produced by each targeted gene (Fig 1D). Expression of the targeted proteins was absent in 3/7 *B2m* KO clones, 4/8 *Jak1* KO clones, and 8/8 *Psmb9* (LMP2) clones. Next, two successfully EMT6 KO clones per targeted gene were selected for further characterization. Specifically, clones 442–5 & 442–6, 450–4 & 450–6, and 454–5 & 454–6 were selected for the *B2m*, *Jak1* and *Psmb9* (LMP2) knockouts, respectively (Fig 1D). For ease, both EMT6 clones for each specific gene deletion will be referred to as clone #1 and clone #2. The *B2m* KO clone 442–5 (clone #1) exemplifies a “failed knockout” as evident by its B2m protein expression (Fig 1D).

### Whole exome sequencing and Sanger sequencing are crucial to confirm the absence of off-target aberrations following CRISPR/Cas9-mediated gene deletion

While western blot is a fast and reliable method to confirm the successful loss of expression of the targeted protein, CRISPR/Cas9-mediated gene deletions can create double-stranded breaks at undesired off-target locations. Here, we used a combination of WES to detect all somatic variants throughout the genome, and Sanger sequencing to identify the exact location and nature of the CRISPR/Cas9-mediated target gene editing.

**Fig 2:**
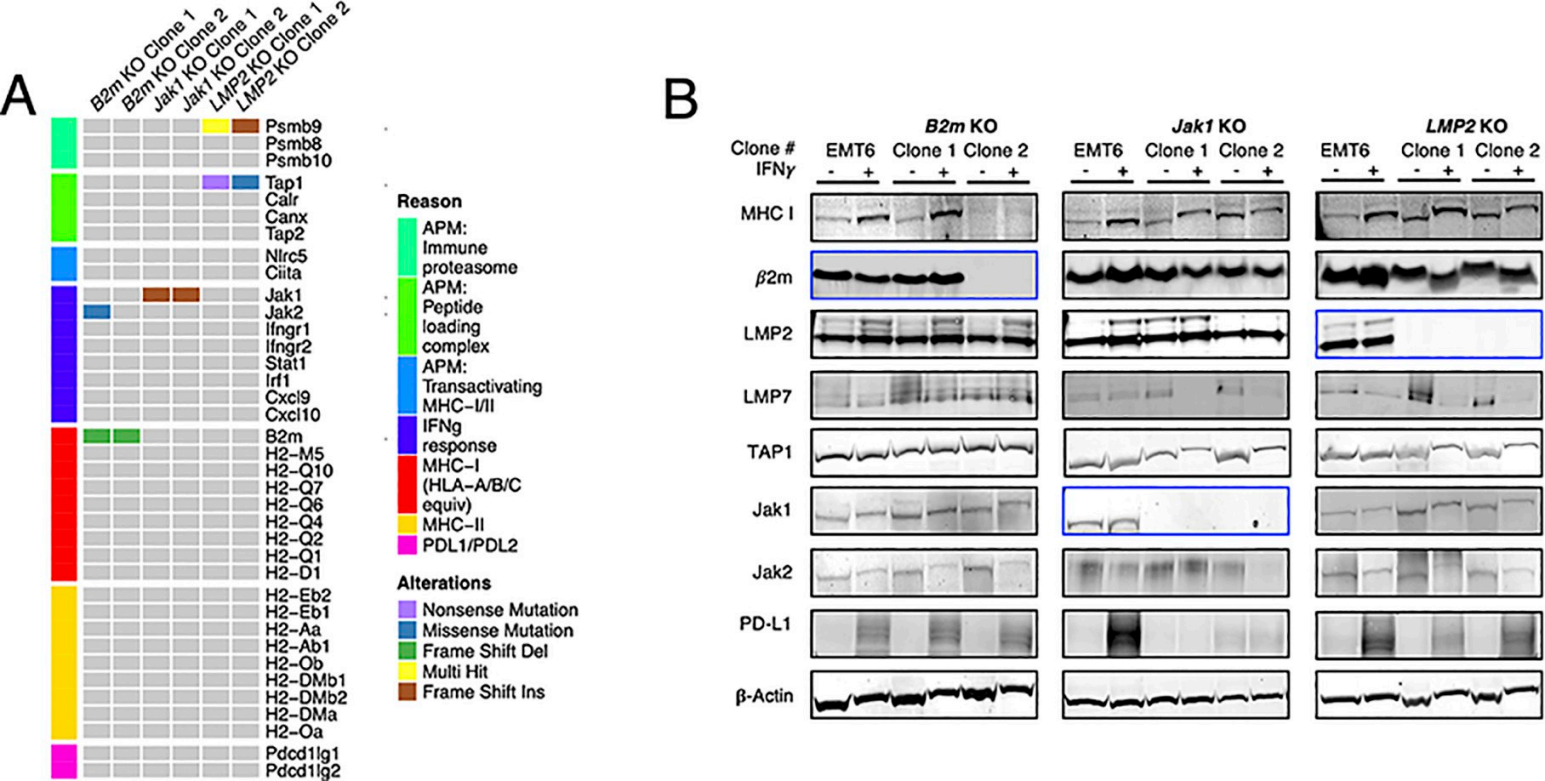
Validation of CRISPR gene knockout single-cell clones. (A) Oncoplot of mutations in selected genes in the generated CRISPR clones. (B) Corresponding western blot quantification of designated proteins in the B2m, Jak1, and LMP2 gene knockout (KO) clones previously incubated for 24 h in the presence or absence of murine IFNγ. Beta-actin was used as loading control. Blue boxes denote protein expression of the KO gene in each corresponding clone. All three KOs were performed once, independently of each other.

For WES analysis, variants were filtered according to the following criteria: (a) depth in tumor cells ≥ 20; (b) variant allele frequency (VAF) in tumor cells ≥ 5%; (c) variant allele depth in tumor cells ≥ 5; and (d) variant allele depth in normal tissue < 5. Variants present in the wild type (wt) EMT6 cell line were subtracted from the CRISPR/Cas9 KO clones’ datasets. To ascertain CRISPR/Cas9 target gene specificity, editing of genes involved in APM, peptide loading complex, MHC class I and II, and IFNγ signaling pathway were examined. As shown in Fig 2A, each CRISPR/Cas9 KO clone contained a gene alteration in the corresponding target gene. There were no off-target gene edits detected in the *Jak1* KO clones, or the *B2m* KO clone #2. However, the *B2m* KO clone #1 contained a missense mutation in the *Jak2* gene occurring at low frequency (10%), which did not result in loss of Jak2 protein expression (Fig 2B). In addition, both *Psmb9* (LMP2) KO clones had gene alterations in the *Tap1* gene occurring at a frequency of 7% (clone #1) and 20% (clone #2), with no effects on protein expression (Fig 2B).

CRISPR/Cas9 KO clones were further analyzed by Sanger sequencing to confirm their purity and identify the exact nature of the indels. The EMT6 *B2m* KO clone #1 displayed three copies of the *B2m* gene, with one copy containing a 1 base pair (bp) cytosine (C) insertion, one copy displaying a 1 bp C deletion, and a wild-type copy with no mutations (Table 1). One can speculate that the wild-type copy is responsible for the expression of B2m protein observed by western blot (Figs 1D and 2B). The *B2m* KO clone #2 displayed a 2 bp deletion on one copy of the gene, and a 324 bp deletion on the other copy, resulting in the full loss of B2m protein expression. Similarly, both the *Jak1* KO clones #1 and #2 lost their Jak1 protein expression due to a 1 bp insertion of adenine (A) or thymine (T), respectively. *Psmb9* (LMP2) KO clone #1 displayed a 10 bp deletion and a 22 bp deletion on the first copy of the LMP2 gene, plus a 1 bp deletion on the second copy of the gene. *Psmb9* KO clone #2 harbored a 1 bp deletion, resulting in the loss of LMP2 protein expression.

**Table 1:** Sequencing results. Summary table of the Sanger sequencing, whole exome sequencing, and western blotting results in EMT6 KO clones. Transcript and protein change columns describe the position and change according to the Human Genome Variation Society (HGVS) nomenclature.

Target gene	Clone #	Chr	Sanger sequencing	WES variant class	Variant allele frequency	Transcript change	Protein change	Target protein expression (+/-)
*B2m*	1	Chr 2	Copy 1: 1 bp insertion of “C” at position chr2:122,150,904; Copy 2: 1 bp deletion of “C” at position chr2:122,150,904; Copy 3: wild type copy	Frame shift insertion	*B2m* = 56% *Jak2* = 10%	c.101del	p.P34Hfs*10	(+)
	2		Copy 1: 2 bp deletion at chr2:122,150,904; Copy 2: 324 bp deletion spanning chr2:122150599–122150922	Frame shift deletion	*B2m* = 100%	c.101del	p.P34Hfs*10	(-)
*Jak1*	1	Chr 4	Copy 1: 1 bp insertion of A at chr4:101,184,444; Copy 2: 1 bp insertion of T at chr4:101,184,444	Frame shift insertion	*Jak1* = 100%	c.375dup	p.V126Cfs*14	(-)
	2		1 bp insertion of T at chr4:101,184,444	Frame shift insertion	*Jak1* = 100%	c.375dup	p.V126Cfs*14	(-)
*Psmb9*	1	Chr 17	Copy 1: 10bp deletion (chr17:34,185,767–34,185,776) and 22 bp deletion (chr17:34,185,810–34,185,831); Copy 2: 1 bp insertion of “T” at chr17:34,185,771	Frame shift insertion	*Psmb9* = 100% *Tap1* = 7%	c.86dup	p.D29Efs*9	(-)
	2		1 bp insertion of T	Frame shift insertion	*Psmb9* = 100% *Tap1* = 20%	c.86dup	p.D29Efs*9	(-)

A, adenine; B2m, beta 2 microglobulin; Bp, base pair; C, cytosine; Chr, chromosome; Jak1, Janus kinase 1; Jak2, Janus kinase 2; Psmb9, proteasome subunit beta type-9; T, thymine; Tap1, transporter associated with antigen processing 1; WES, whole exome sequencing.

To validate the absence of off-target protein deletion following *B2m*, *Jak1*, and *Psmb9* knockouts, western blots were performed on proteins playing a major role in APM machinery and the IFNγ signaling pathway. To this end, western blots were conducted using 100 μg of whole-cell protein lysate against MHC I, B2m, LMP2, LMP7, TAP1, Jak1, Jak2, and PD-L1 proteins after a 24 h exposure to 100 ng/mL of murine IFNγ or media control (Fig 2B). Wild-type EMT6 cells expressed high baseline levels of B2m, LMP2 and TAP1, moderate levels of MHC class I, Jak1 and Jak2, low levels of LMP7, and no detectable PDL1 expression. IFNγ exposure induced PD-L1 expression and upregulated MHC I proteins (Fig 2B). Compared to the wt EMT6, successful deletion of *B2m* and *Psmb9* (LMP2) did not impact the expression of other proteins examined. However, successful deletion of *Jak*1 abrogated PD-L1 protein expression. The lack of PD-L1 expression was not attributed to CRISPR/Cas9 off-target gene deletions since its encoding gene *Pdcd1lg1* remained intact (Fig 2A). Collectively, the sequencing and western blot results confirmed that the *B2m* KO clone #2, and both *Jak1* and *Psmb9* (LMP2) KO clones were successfully knocked out.

For the subsequent clone validation process, one EMT6 clone per target gene was selected, namely the *B2m* KO clone #2 (442–6), *Jak1* KO clone #1 (450–4), and *Psmb9* KO clone #1 (454–5).

### Quantification of protein expression *in vitro* and *in vivo* is necessary to validate the functionality of CRISPR/Cas9 clones

**Fig 3:**
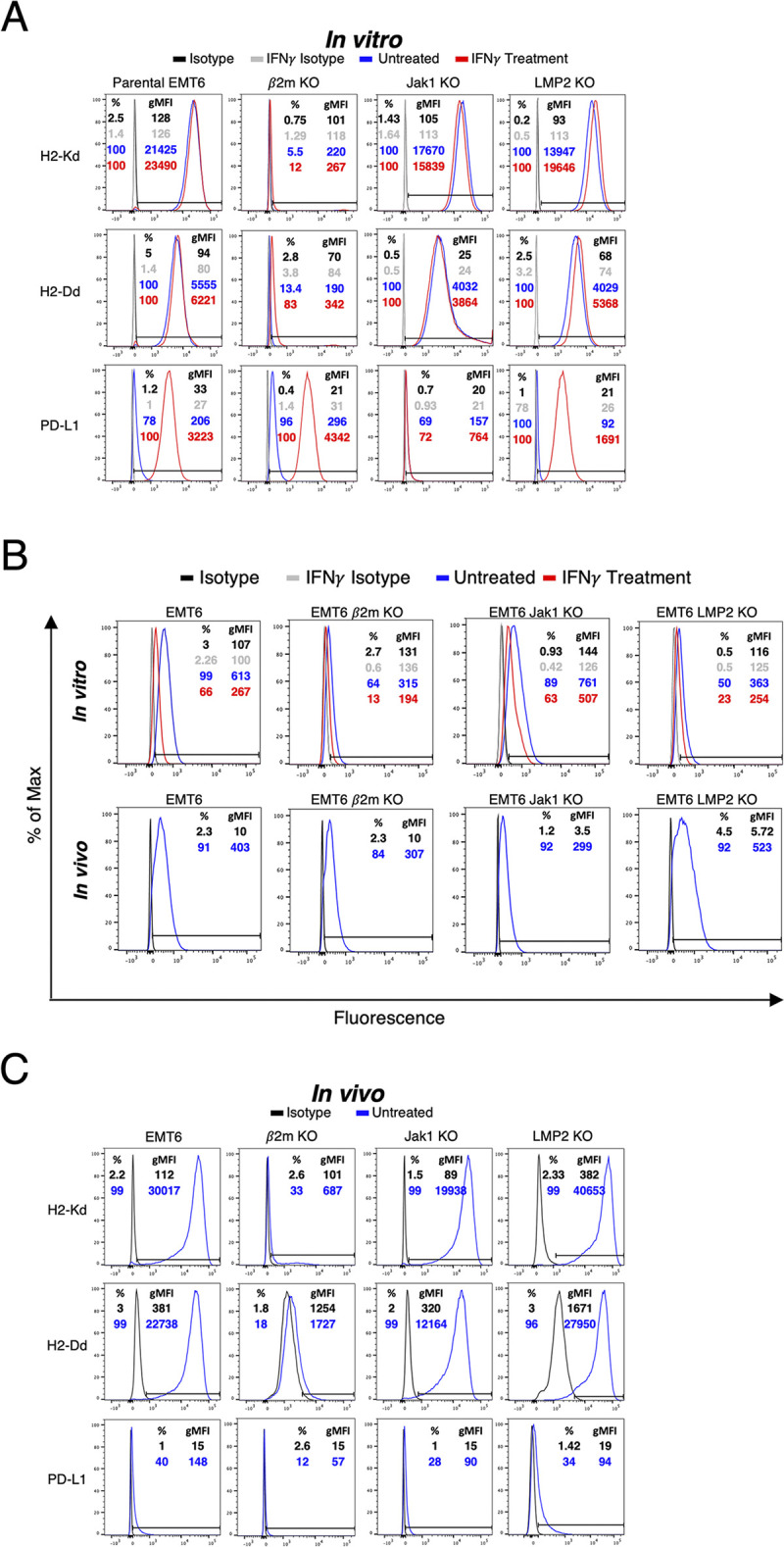
Effects of knocking out β2m, *Jak1*, and LMP2 on the expression of immune-related cell surface markers *in vitro* and *in vivo*. (A) Representative histograms of *in vitro* cell surface expression of MHC class I haplotypes (H2-K^d^ and H2-D^d^) and PD-L1 in EMT6 wild type (wt) cell line and corresponding KO cell lines on untreated cells compared to staining isotype controls and IFNγ-treated cells. (B) Representative histograms of cell surface expression of IFNγRI *in vitro* (top panel) on untreated cells compared to staining isotype controls and IFNγ-treated cells, and *in vivo* (lower panel) on untreated EMT6 wt and CRISPR KO clones compared to staining isotype controls. (C) Representative histograms of *in vivo* cell surface expression of MHC class I haplotypes (H2-K^d^ and H2-D^d^) and PD-L1 in untreated EMT6 wt tumors and designated single gene-deleted EMT6 KO tumors compared to isotype controls. Insets denote percentage of positive cells and geometric mean fluorescence intensity (gMFI) for the designated protein. *In vitro* data are representative of 2 independent experiments with 3 replicates per experiment. *In vivo* data are representative of 2 independent experiments with 4–7 mice per experiment. KO cell lines: *B2m* KO clone #2 (442–6), *Jak1* KO clone #1 (450–4), and *Psmb9* KO clone #1 (454–5).

To further validate and extend our findings at the protein level, wt EMT6 cells were compared to the KO clones for cell surface expression of MHC class I (H2D^d^ and H2K^d^), PD-L1, and IFNγR1 by flow cytometry in the absence or presence of IFNγ *in vitro* exposure. As expected, deletion of *B2m* led to complete loss of H2D^d^ and H2K^d^ proteins without affecting PD-L1 regardless of IFNγ exposure. Deletion of *Jak1* led to severe loss of PD-L1 expression with no effect on H2D^d^, H2K^d^; PD-L1 expression could not be recovered by IFNγ exposure. Deletion of *Psmb9* (hence LMP2 protein) had no effect on the cell-surface expression of PD-L1, H2D^d^, H2K^d^ (Fig 3A). In addition, deletion of *B2m or Psmb9* but not *Jak1* let to a moderate decrease in the *in vitro* expression of IFNγRI (Fig 3B). Successful B2m deletion was also validated in MC38 cells, with clone #3 selected for subsequent in vivo studies (S1 Fig in S1 File).

Next, wt EMT6 cells and KO clones were implanted into syngeneic Balb/C female mice via subcutaneous injection in the mammary fat pad using either 2.5 x 10^5^ or 5 x 10^5^ cells per implant (S2 Fig in S1 File). Starting on day 7 post-tumor implant, tumor volumes were measured twice weekly and calculated as (length2 × width)/2. We confirmed that the tumor growth rate of EMT6 wt and the KO clones remained similar (S2 Fig in S1 File). When their volume reached 1000 mm^3^, wt and KO tumors were excised and examined by flow cytometry to validate in vivo tumor cell surface expression of H2D^d^, H2K^d^, PD-L1, and IFNγRI. *In vivo* expression of IFNγRI was unaffected by any of the single-gene deletions described here (Fig 3B). In vivo expression of H2D^d^, H2K^d^ was similar to that observed *in vitro*, with PD-L1 expression on the tumor cell surface remaining unremarkable (Fig 3C). Analysis of MC38 B2m KO cells demonstrated absence of B2m protein in vitro and in vivo (S1 Fig in S1 File).

Tumors from wild-type EMT6 cells have been reported to harbor large areas of necrosis and to be rich in collagen [28,29]. Immunotherapies targeting either areas of necrosis or intra-tumoral collagen in murine tumor models, including EMT6, have previously been reported as efficacious alternatives to ICB [28,29]. As an additional validation step, we examined tumor necrosis by hematoxylin and eosin (H&E), and performed trichrome staining in order to quantify collagen levels (S2 Fig in S1 File). Quantification of necrosis and collagen levels in tumors of similar size using the freely accessible QuPath v0.2.3 software [30] revealed no significant changes in necrosis upon deletion of any of the target genes (S2 Fig in S1 File). Similarly, no significant differences in collagen levels between tumors originated from EMT6 wt cell line and the *Jak1* or *Psmb9* KO clones were observed. However, tumors from *B2m* KO cells displayed a 2-fold increase in collagen content relative to EMT6 wt tumors (S2 Fig in S1 File).

**Fig 4:**
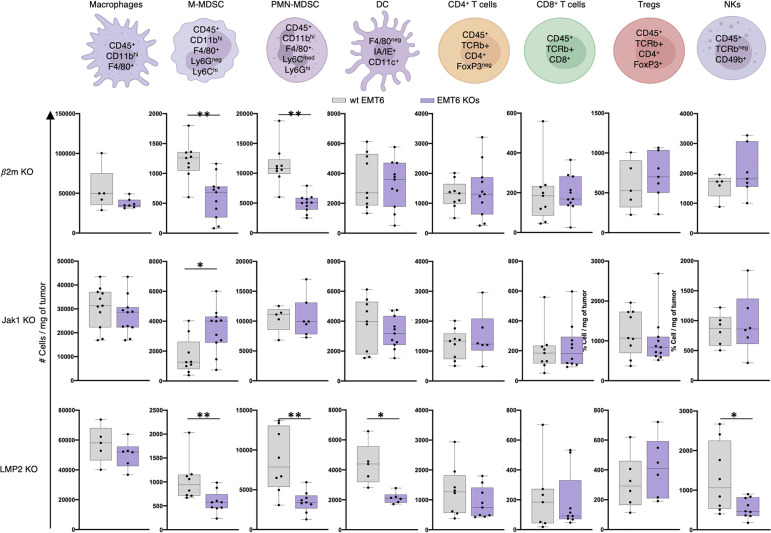
Effects of β2m, *Jak1*, and LMP2 single-gene deletion on the intra-tumoral immune cell landscape. Quantification of macrophages, myeloid derived suppressor cells (MDSC) of mononuclear (M-MDSC) or polymorphonuclear origin (PMN-MDSC), dendritic cells (DC), CD4^+^ T-lymphocytes (CD4), regulatory CD4^+^ T-lymphocytes (Tregs), CD8^+^ T-lymphocytes (CD8), and natural killer cells (NK) per milligram of individual EMT6 wt and designated gene KO tumors, identified by the designated phenotype. KO cell lines: *B2m* KO clone #2 (442–6), *Jak1* KO clone #1 (450–4), and *Psmb9* KO clone #1 (454–5).Data are representative of 2 independent experiments with 4–7 mice per experiment. Box plots denote values from each tumor, with median±SD. Tumors were analyzed when their volume reached ~1000 mm^3^. Two-tailed t-test, * = p < 0.05, ** = p < 0.01, *** = p < 0.001, **** = p < 0.0001.

Next, using flow cytometry, we interrogated the effect of tumor cell target gene deletion on the immune cell composition in the tumor microenvironment. Tumors from wt EMT6 or MC38 cell lines and the corresponding KO clones were harvested when tumor volume reached approximately 1000 mm^3^. Tumor processing and antibody staining are detailed in the Methods section. Cell populations were identified using gating strategies shown in S4 Fig in S1 File. Compared to wt tumors, the absence of B2m, Jak1 and LMP2 expression on tumor cells resulted in a significant reduction of monocytic myeloid-derived suppressor cells (M-MDSC) in the TME. In addition, loss of expression of tumor cells B2m and LMP2, but not Jak1, led to a significant decrease of polymorphonuclear myeloid-derived suppressor cells (PMN-MDSC) infiltrates in the TME (Fig 4). Significant decrease in both MDSC populations was also observed in MC38 B2m KO tumors (S1 Fig) in S1 File. Moreover, the absence of LMP2 expression on EMT6 tumor cells significantly hindered the presence of dendritic cells (DCs) and natural killer (NK) cells in the TME compared to wt EMT6 tumors (Fig 4). The absence of B2m, Jak1 and LMP2 expression on EMT6 tumor cells did not impact the infiltration of macrophages, effector CD4^+^ or CD8^+^ T cells, or CD4^+^ regulatory T cells (Tregs). However, lack of B2m in MC38 tumor cells resulted in a significant decrease in tumor-infiltrating macrophages, DCs and CD8^+^ T cells, without appreciable impact in CD4^+^, Tregs, or NK lymphocytes (S1 Fig in S1 File).

### Validated murine tumors generated from KO clones were refractory to αPD-1 and αPD-L1 therapies

**Fig 5:**
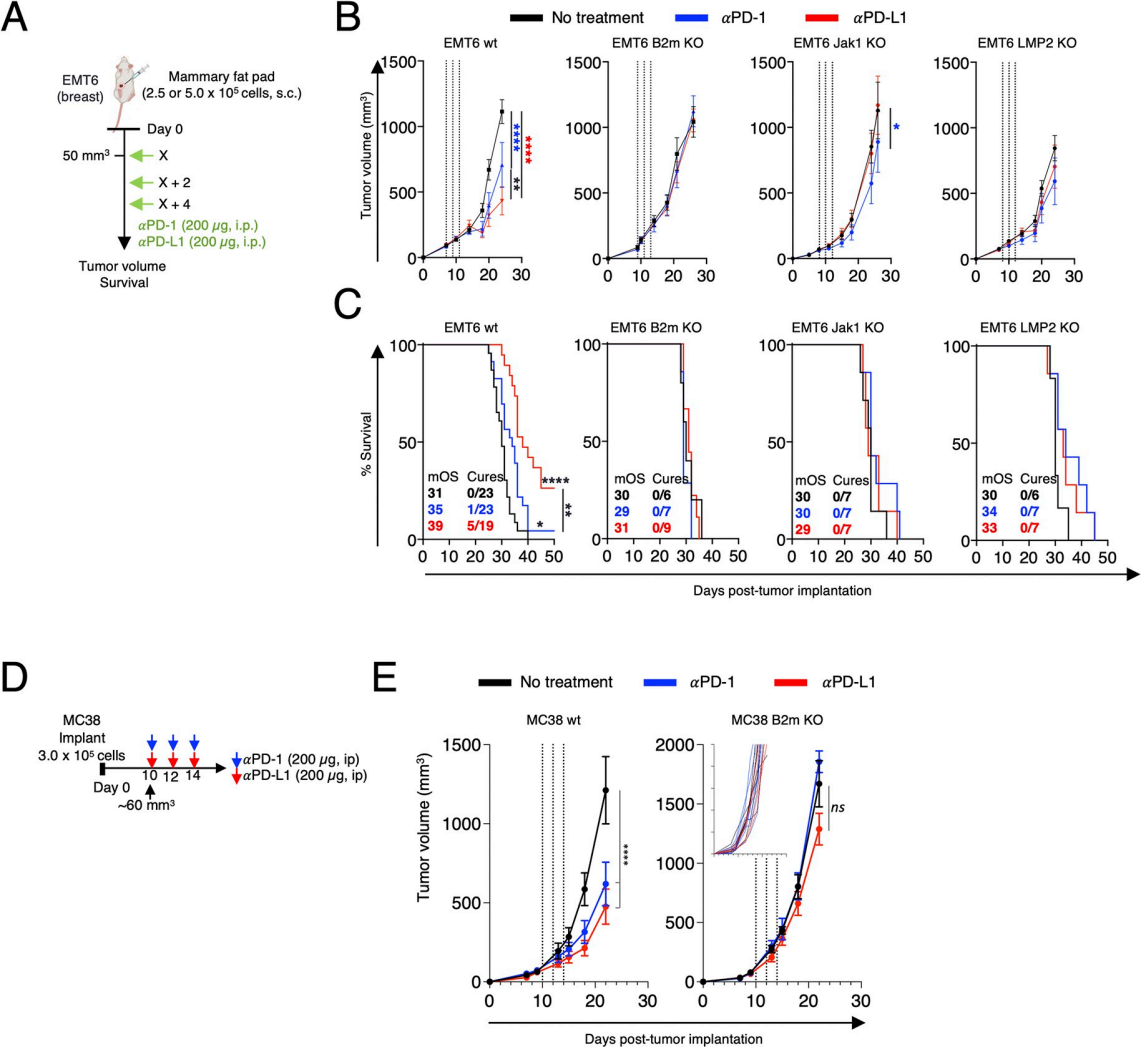
Gene knockout tumor models are unresponsive to immune checkpoint inhibitor therapies. Generic treatment schedule for all EMT6/EMT6 knockout (KO) (A) and MC38/MC38 B2m KO (C) tumor experiments. Arrows indicate days post tumor implant (day 0) when mice received αPD-1 or αPD-L1. Treatment was initiated when tumors reached an average volume of 50 mm^3^. (EMT6/EMT6 KO) or 60 mm^3^ (MC38/MC38 B2m KO). Graphs show (B) representative tumor growth curves (mean ± SEM) and (C) survival of EMT6 wt and gene KO tumors (n = 6-7/group) treated as depicted in the schematic in Fig 5A. Insets denote median overall survival (mOS) and number of mice without palpable tumors per treatment group at the time of study termination (day 50). (E) Graphs show tumor growth curves (mean ± SEM) of MC38 wt and B2m KO tumors (n = 5-7/group) treated as depicted in the schematic in Fig 5D. Inset denotes tumor growth in individual mice implanted with MC38 B2m KO cells and treated as depicted in Fig 5D. Black dashed lines depict days of αPD-1 or αPD-L1 doses. Tumor growth curves and corresponding survival of each KO tumor model were performed once independently of each other. The corresponding wt EMT6 data were performed three times independently of each other (data pooled). KO cell lines: EMT6 *B2m* KO clone #2 (442–6), EMT6 *Jak1* KO clone #1 (450–4), EMT6 *Psmb9* KO clone #1 (454–5), and MC38 B2m KO clone #3. Ordinary two-way ANOVA with Tukey’s multiple comparisons test (B), or two-tailed Log-rank (Mantel–Cox) (C). * = p < 0.05, ** = p < 0.01, *** = p < 0.001, **** = p < 0.0001.

The EMT6 breast carcinoma is a poorly immunogenic tumor model that has a moderate response to αPD-1 and αPD-L1 therapies [31,32]. To confirm *in vivo* that the KO clones were irresponsive to ICB therapy, wt EMT6 cells and its KO clones were subcutaneously implanted into the mammary fat pad of syngeneic Balb/C female mice. When tumors reached a volume of ~50 mm^3^, mice were left untreated or received αPD-1 or αPD-L1 (200 μg, *i*.*p*.) administered every other day for a total of three doses (Fig 5A).

Using this treatment schedule, both αPD-1 and αPD-L1 treatments led to significant tumor growth control and improved overall survival in mice harboring wt EMT6 tumors (Fig 5B and 5C). The median overall survival of the wt EMT6 increased from 31 days to 35 days and 39 days following αPD-1 and αPD-L1 therapy, respectively. Notably, 1 out of 23 mice (4.3%) that received αPD-1 and 5 out of 19 mice (26.3%) that received αPD-L1 were devoid of palpable tumors at the time of study termination (S3 Fig in S1 File). In contrast, tumors from the B2m/Jak1/LMP2 KO single-cell clones did not respond to either αPD1 or αPDL1 treatment. Similar studies in mice implanted with MC38 B2m KO cells indicated abrogated tumor response to either immune checkpoint inhibitor, contrasting with a significant decrease in tumor growth observed in mice harboring MC38 wt tumors (Fig 5D and 5E).

## Discussion

Identifying novel therapies for patients refractory to immune checkpoint blockade is an area of high clinical interest, as ICB is often the last line of therapy available. Research has been conducted to identify predictive biomarkers of ICB response/resistance, including tumor mutational burden, microsatellite instability and mismatch repair deficiency, circulating protein and DNA, tumor-infiltrating lymphocyte count, and gene aberrations [33]. Gene mutations in the antigen processing machinery and in the IFNγ signaling pathway are well-documented hallmarks of resistance to PD-1 or PD-L1 blockade in multiple cancers with potential consequences for patient response to immunotherapies at large, including adoptive T cell therapy and vaccines [12,14,16,23]. Our analysis of 33 publicly available solid cancer datasets from TCGA revealed that while the frequency of mutations in the aforementioned pathways remains relatively low for each single gene, mutations were observed in multiple genes within a pathway and in all tumor types examined. These findings are in agreement with previously reported gene mutation analysis from melanoma [34,35], lung [36], and colon cancer [37] lesions from patients who developed resistance to ICB.

As a proof of concept, *B2m*, *Jak1*, and *Psmb9* single gene KOs were generated using the murine EMT6 breast tumor model to promote PD-1 or PD-L1 ICB resistance. B2m deletion was also examined in the MC38 murine colon cancer model. Traditionally, targeted gene knockdown has been achieved by RNA interference. However, the resulting knockdown is often incomplete, greatly varies between experiments, leads to off-target gene effects, and only provides temporary inhibition of gene expression [38]. Instead, transcription activator-like effector nucleases (TALEN), zinc finger nucleases (ZFN), and CRISPR/Cas9 can be employed to achieve permanent double-strand breaks in DNA and complete gene deletion. Here, we employed the CRISPR/Cas9 technology to produce the single-cell KO clones. Compared to TALEN and ZFN, CRISPR/Cas9 does not rely on protein-based binding to DNA but uses short guide RNA (sgRNA) sequences to drive site specific double-strand breaks in a more time efficient and cost-effective manner [38,39]. While one study reported that CRISPR/Cas9 is less specific than TALEN and ZFN [40], the majority of published reports describe no detectable off-target cleavage caused by CRISPR/Cas9 other than that caused by the lack of sgRNA specificity [41].

Here, sgRNAs were identified using the sgRNA Scorer 2.0 algorithm and incorporated into the commercially available BsmBI-digested Lenti-CRISPRv2GFP plasmid encoding both the Cas9 protein and a green fluorescence protein (GFP) to allow for the positive selection of successfully transfected cells. The well-established non-viral delivery vehicle consisting of lipofectamine resulted in transfection efficiency varying between 7% and 29%. Several factors could be optimized to increase transfection efficiency, including the ratio of plasmid DNA to lipofectamine, the incubation time, or the number of cells per well. Alternatively, transfection reagents compatible with serum-containing medium could be used, such as the ViaFect^TM^ transfection reagent kit. Different CRISPR delivery systems are also available, including physical delivery (e.g., microinjection and electroporation), viral vectors (e.g., adeno-associated virus (AAV), adenovirus, and lentivirus), and non-viral vectors (e.g., lipid nanoparticles, cell penetrating peptides, gold nanoparticles), which are reviewed elsewhere [41]. Each of these systems currently presents its own drawbacks. Briefly, physical microinjection of the plasmid construct into the cytoplasm yields efficiencies approaching 100% but remains labor intensive. Electroporation is most suited for bacterial cells since mammalian cells are sensitive to applied voltages. AAV and lentivirus can integrate into the host DNA, resulting in a persistent source of the CRISPR DNA, increasing the likelihood of off-target cutting incidence. In addition, the host cell becomes more immunogenic upon lentivirus and adenovirus viral transfection.

Methodologies are being developed to detect off-target CRISPR/Cas9 editing and are reviewed elsewhere [25,42]. These include Genome-wide, Unbiased Identification of Double-stranded breaks Enabled by Sequencing (GUIDEseq), Selective Enrichment and Identification of Adapter-Tagged DNA Ends by Sequencing (SITE-seq), Circularization In Vitro Reporting of Cleavage Effects by Sequencing (CIRCLE-seq), and Discovery of In Situ Cas Off-targets and Verification by Sequencing (DISCOVER-Seq). Here, WES was complemented with Sanger sequencing to detect any CRISPR-induced off-target mutations. No off-target gene edits were detected in the *Jak1* KO clones, or the *B2m* KO clone #2. However, both *Psmb9* (LMP2) KO clones had gene alterations in the *Tap1* gene occurring at a frequency of 7% (clone #1) and 20% (clone #2), with no effects on protein expression. Future improvement in the currently available computational tools may allow for a more thorough sequencing analysis of a larger number of sgRNAs, including a better prediction of potential off-target sites, thereby preventing them.

The successful loss of expression of the targeted protein in the *B2m*, *Jak1*, or *Psmb9* KO clones, and their effects on other proteins playing a major role in APM machinery (TAP1, LMP7, H2D^d^, and H2K^d^) and the IFNγ signaling pathway (IFNγRI, Jak2, and PDL1) were quantified by western blot and/or flow cytometry and compared to the wt cells. As expected, deletion of *B2m* in either EMT6 or MC38 led to complete loss of H2D^d^ and H2K^d^ expression, preventing tumor recognition and elimination by CD8^+^ T cells [21]. As demonstrated by western blotting, deletion of *Jak1* abrogated PD-L1 expression, devoiding the tumor cell of a primary target of immune checkpoint blockade [23]. Despite validated complete deletion of the *Jak1* gene, flow cytometric analysis indicated minor cell-surface PD-L1 expression in vitro at baseline in the selected EMT6 Jak1 KO clone, similar to that observed in wt EMT6 cells. In contrast to wt cells, IFNγ exposure did not induce appreciable change in the population of Jak1 KO cells expressing PD-L1 in vitro, albeit a minor increase in expression on a per-cell basis. No appreciable PD-L1 expression was observed in Jak1 KO tumor cells in vivo, where blockade of unspecific binding of the staining antibody is used. Collectively, this apparent discrepancy in the in vitro findings may be due to unspecific binding of the anti-PD-L1 antibody used in flow cytometric analysis. Deletion of *Psmb9* did not impact the expression of any of the proteins examined. Nevertheless, the absence of LMP2 is expected to prevent the degradation of endogenous tumor antigens in the cytosol, thereby averting their loading onto MHC class I and their presentation to cognate T lymphocytes [22]. Further studies beyond the scope of the methodology described here are required to confirm the loss of proteasomic activity using commercially available fluorometric proteasome activity assay kits.

The tumor microenvironment harbors cancer cells in a milieu containing tumor-infiltrating immune cells, blood vessels, fibroblasts, and a collagen rich extracellular matrix. Among the tumor types that respond poorly to ICB, colorectal, pancreatic, and breast cancers are characterized by a collagen-dense extracellular matrix [43]. Collagen acts as a physical barrier to immune cell infiltration and suppresses anti-tumor immune activity, contributing to PD-1 or PD-L1 ICB resistance [44]. In addition, tumor necrosis is an indicator of poor prognosis in kidney, lung and breast cancer malignancies [45]. In preclinical studies, immunotherapies targeting either tumor necrosis or intra-tumoral collagen have been shown to suppress ICB-sensitive tumors such as EMT6 and to be efficacious alternatives to ICB [28,29]. Quantification of necrosis and collagen levels revealed no significant differences between wt EMT6 tumors and the *Jak1* or *Psmb9* KO tumors. However, tumors from *B2m* KO cells displayed a 2-fold increase in collagen content relative to EMT6 wild-type tumors, suggesting that tumors harboring *B2m* deficiencies may benefit from therapies targeting collagen.

During cancer progression, antigens released by dying tumor cells are presented by antigen-presenting cells (APCs) to CD4^+^ or CD8^+^ T cells, resulting in immune activation and a cascade of events that are the foundation of cancer immunity [46]. The tumor microenvironment is a dynamic milieu reflecting a constantly evolving immune composition in response to a spectrum of signals from tumor cells, stromal cells, and other elements of the TME [47]. Notably, the presence in the TME of IFNγ and other cytokines produced by activated immune cells, as well as immune-attracting chemokines and tumor secretion of soluble factors, are well-established elements impacting tumor immune infiltration [48]. To assess the potential immune consequences of tumor cell *B2m*, *Jak1*, and *Psmb9* gene deletion, immune infiltrates in advanced wild-type and gene KO tumors were examined. Compared to wt EMT6, the absence of B2m or LMP2 protein expression on tumor cells resulted in a significant reduction of M-MDSCs and PMN-MDSCs in the TME, with Jak1 deletion only decreasing the former. The absence of tumor LMP2 expression significantly hindered the frequency of DCs and NK cells in the TME compared to wt EMT6 tumors. However, the absence of B2m, Jak1 or LMP2 expression on EMT6 tumor cells did not impact the frequency of macrophages, effector CD4+ or CD8^+^ T cells, or Tregs in the TME. Similar effects on tumor infiltration of both MDSC populations, effector CD4^+^ T cells, CD4^+^ Tregs, and NK cells were observed in MC38 B2m KO tumors. However, and in contrast to EMT6 B2m KO tumors, deletion of B2m on MC38 tumor cells resulted in significant reduction of macrophage, DC, and CD8^+^ T cells in the TME. In future studies, it will be important to examine if baseline immune infiltration correlates with these effects as MC38 tumors have significant CD8 infiltration whereas EMT6 tumors do not. Investigation of the immune effects elicited by ICB in these KO tumors may provide insight on the potential immune impact of adding ICB to combination therapies for the treatment of ICB-refractory patients. Additional studies beyond the scope of this report will be necessary to allow for a deeper understanding of the specific TME alterations driving immune infiltration in the KO tumors. Generation of additional KO clones, including in other ICB-responsive tumor models such as MB49 (bladder) and B16-F10 (melanoma) may provide additional mechanistic insight. In addition, it cannot be ruled out that these observations could differ at a different stage during tumor evolution, particularly in smaller tumors.

Syngeneic mice bearing breast carcinoma EMT6 tumors attained moderate tumor suppression, including some complete responses to αPD-1 (4.3%) and αPD-L1 (26.3%). These results are in agreement with previous studies [31,32], and reflect the overall response rate observed in breast cancer patients [49,50]. In contrast, mice bearing tumors from the *B2m* (EMT6 and MC38) and *Jak1/LMP2* (EMT6) KO single-cell clones did not respond to either αPD1 or αPDL1 treatment. Future studies investigating the impact of ICB and other immunotherapies in immune infiltration and function in the TME in these KO tumor models will be important to better understand the impact of these tumor-cell intrinsic APM/IFNγ pathway defects in treatment response, potentially shedding light on alternative therapies for patients unresponsive to ICB. PD-1/PD-L1 blockade is being evaluated in combination therapies, including the use of chemotherapeutics, small molecule inhibitors (e.g., PARP inhibitors), and alternative immunotherapeutics [49,51]. The development of these and additional KO tumor models will also allow exploring the potential of novel treatements beyond ICB, such as IL-15 and IL-12 immunocytokine therapies, where the presence of these tumor-cell intrinsic gene defects may add clinical benefit via NK cells.

The presented method does have some limitations. While the sgRNAs used here showed high targeting efficiency, CRISPR-Cas9-mediated gene deletions might unintendingly affect overlapping genes. As a result, one specific genomic alteration spot targeted by the gRNA might perturb phenotypes that are affected by that particular genome location. In addition, syngeneic tumors were generated by subcutaneous implantation of tumor cell lines generated from single cell clones, which deviates from the complex polyclonal tumor cell composition observed in patients. While these tumors do not truly capture the TME complexity observed in humans, they constitute well-defined models that can be used to identify promising combination therapies for patients harboring these gene defects. The immune composition of the TME may be time and tumor volume dependent; thus a more comprehensive evaluation requires the analysis of the immune infiltrate at different time points during tumor evolution.

To conclude, the methods presented here open up the possibility of systematic study of gene deletions promoting PD-1 or PD-L1 ICB resistance in murine tumor models, facilitating the discovery of alternative treatment options and a deeper understanding of the immune consequences of tumor mutations, with potential clinical implications.

### Ideas and speculation

One can speculate that the deletion of tumor B2m, LMP2, or Jak1 impacts cytokine and chemokine gradients in the TME given the altered and differential immune cell infiltration observed in these KO tumors relative to each other and to wt tumors. Single gene deletions are likely to promote unique tumor-immune infiltrate profiles depending on the tumor model being used. In addition, syngeneic tumors were generated by subcutaneous implantation of tumor cell lines generated from single cell clones as opposed to a polyclonal tumor cell population. Thus, one can speculate that polyclonal tumors harboring various degrees of B2m, LMP2, or Jak1 mutations may differ on their immune composition relative to our observations. Moreover, the impact of each gene deletion on the tumor immune composition may evolve with tumor development and the type of tumor.

## Materials and methods

### Tumor cell line

The EMT6 cancer cell line (RRID:CVCL_1923), which originates from the Mus musculus species of the female sex, causes a malignant neoplasm in the mammary gland. EMT6 were cultured in Waymouth’s MB 752/1 medium supplemented with 2 mM L-glutamine, 15% (v/v) heat inactivated fetal bovine serum (FBS), and 1% (v/v) penicillin−streptomycin. Cells were grown at 37°C and 5% CO2. All cells were free of mycoplasma as determined by MycoAlert Mycoplasma Detection Kit (Lonza, Walkersville, MD) and were used at low passage numbers (between P3 and P5).

### Rodents

Animal studies were reviewed and approved by the NIH Institutional Animal Care and Use Committee (Protocol LTIB-038), and studies were performed in an Association for Assessment and Accreditation of Laboratory Animal Care-accredited animal facility of the National Institutes of Health (NIH). Six- to eight-week-old female test naive Balb/c mice (RRID:IMSR_APB:4790) were obtained from the NCI Frederick Cancer Research Facility (Frederick, MD). Five to six animals were co-housed in microisolator cages under pathogen-free conditions using a 12h:12h day/night cycle, in rooms maintained at 72°F ± 2°F and 30–70% relative humidity. Mice were euthanized by cervical dislocation.

### TCGA data analysis

Publicly available mutation data from The Cancer Genome Atlas (TCGA) were retrieved as Mutation Annotation Format (MAF) files using the *TCGABiolinks* R package (version 2.20.1) [26]. Single nucleotide polymorphisms (SNPs) and small insertions/deletions (Indels) called by ‘mutect2’ were downloaded and summarized for all 33 cancer types. Human gene symbols were manually curated into five categories: antigen presentation machinary (APM, 9 genes), major histocompatibility complex (MHC, 8 genes), checkpoint inhibitor ligands (7 genes), IFNγ pathway (9 genes), and immune-associated markers (9 genes). Mutations were restricted to non-silent mutations, defined as the 12 variant classifications with “High” or “Moderate” impact reported by Variant Effect Predictor (VEP) [27]. (Refer to https://useast.ensembl.org/info/genome/variation/prediction/predicted_data.html for a complete list.) The number of samples with one or more mutations in each gene were tallied, and this was repeated for all cancer types independently.

### CRISPR/Cas9 plasmid generation to knockout *B2m*, *Jak1* and *Psmb9*

Multiple candidate guide RNAs targeting *B2m*, *Jak1*, and *Psmb9* were identified using the sgRNA Scorer 2.0 algorithm [52]. Guide RNAs were *in vitro* transcribed, complexed with Cas9 protein, and transfected into P19 cells [53]. DNA was extracted using Quick Extract (Lucigen, Middleton, WI) and PCR amplicons between 220 to 450 bp in size, encompassing the target sites, were generated using the NEB Q5 HotStart polymerase (New England Biolabs, Ipswich, MA). Products were sequenced on the Illumina MiSeq (San Diego, CA) using the Nano V2 300 cycle (150 x 2 format). Data were analyzed using an in-house developed pipeline (https://github.com/rajchari2/ngs_amplicon_analysis) as previously described [53]. Candidates 1756 (*B2m*), 1780 (*Jak1*), and 1810 (*Psmb9*) were selected for further experiments. Oligonucleotides corresponding to these guides (S1 Table in S1 File) were annealed and ligated into BsmBI-digested Lenti-CRISPRv2GFP plasmid [54] and transformed in Lucigen Endura cells (Lucigen, Middleton, WI). Twenty ng of gDNA from single-cell derived populations were used in a Q5 PCR. PCR products were then gel extracted and TOPO cloned using the Zero Blunt TOPO PCR cloning kit (Thermo Fisher Scientific, Pleasanton, CA). Cloned products were subsequently transformed in NEB 5 Alpha cells (New England Biolabs, Ipswich, MA), multiple single colonies were amplified in 5 mL cultures and plasmids were purified using the Genejet Miniprep kit (Thermo Fisher Scientific, Pleasanton, CA). Plasmids were then Sanger sequenced using the SP6 sequencing primer and data were then analyzed using Geneious Prime 2020.1.2 software (Dotmatics, Boston, MA).

### Plasmid amplification, purification, and characterization

Glycerol stocks of NEB 5 Alpha cells were streaked on a LB-agar plate containing ampicillin and colonies were grown overnight at 37°C. A starter culture was obtained by gently placing a single colony into 5 mL of LB Agar containing 50 μg/mL ampicillin overnight at 37°C. A volume of 2.5 mL of the starter culture was incubated with 100 mL fresh LB culture supplemented with 50 μg/mL ampicillin for 8 h into a bacteria shaker. Each culture was collected into 50 mL falcon tubes and pelleted down at 4000xg for 10 min. Pellets were kept at -20°C until further processing. Plasmids were purified from bacteria pellets using a PureLink HiPure plasmid maxiprep kit (Thermo Fisher Scientific, Pleasanton, CA) following the manufacturer’s protocol. The concentration of purified plasmids was determined by UV–vis spectroscopy using a NanoDrop One instrument, and the Absorbance ratios at 260:280 and 260:230 were recorded.

### Sanger sequencing

To confirm their purity, plasmids were sequenced by Sanger sequencing using the hU6-F primer (5’-GAGGGCCTATTTCCCATGATTCC-3’).

### Cell transfection with CRISPR constructs

When wt cells reached their third passage, their conditioned media supernatant was collected and filtered using 0.22 μm vacuum driven Steriflip® tubes (EMD Millipore, Burlington, MA) for later use. Cells were seeded into 6-well plates using 50,000 cells per well. When cells reached 70% confluency, cells were transfected with their respective CRISPR/Cas9 plasmid constructs using the Lipofectamine® 3000 transfection kit (Thermo Fisher Scientific, Pleasanton, CA) following the manufacturer’s recommendation. For each well transfected, 230 μL of OptiMEM was first mixed with 20 μL of Lipofectamine. Separately, 230 μL of OptiMEM was mixed with 10 μg of plasmid constructs and 20 μL of P3000 reagent. The two reactions were combined and incubated for 15 min at room temperature. A volume of 250 μL of the final mixture was incubated with wt EMT6 cells and complemented with 500 μL of OptiMEM for 2½ h before adding 1.5 mL of cell culture media. Forty-eight to 72 h later, cells were trypsinized (0.25% Trypsin-EDTA), washed twice with phosphate buffer saline (PBS) by spinning cells down at 400xg for 4 min, and resuspended in PBS. Successfully transfected cells were positively selected using a SONY MA900 cell sorter (Sony, San Jose, CA) based on their positive GFP expression, and individually seeded (single cell) onto a 96-well plate containing a 1:1 solution of fresh cell culture media and conditioned media from wt EMT6 cells (ATCC, Gaitherburg, MD). Cells were allowed to replicate for 2 weeks at 37°C and 5% CO2. Ten clones from each gene knockout were randomly selected and transferred onto 12-well plates for further expansion. When cell confluency reached 90%, clones were expanded onto T-150 culture flasks and cell stocks were prepared (~3–5 million cells in heat inactivated FBS supplemented with 5% (v/v) DMSO) and frozen at -80°C until use.

### Western blot protein quantification

Wt EMT6 cells and their subsequent CRISPR/Cas9 knockout clones were seeded (500,000 cells/well) onto 6-well plates and incubated for 24 h prior to treatment with 100 ng/mL of murine IFNγ for another 24 h period. Cells were then dissociated with 0.25% Trypsin-EDTA, washed twice with PBS, and lysed using radioimmunoprecipitation assay buffer (RIPA) supplemented with 1X protease and phosphatase inhibitor. Protein lysate concentration was quantified using the Pierce™ BCA Protein Assay kit (Thermo Fisher Scientific, Pleasanton, CA) following the manufacturer’s recommendation. One hundred μg of cell protein lysate was supplemented with Bolt™ Lithium dodecyl sulfate (LDS) sample buffer (4X) and denatured at 95°C for 5 min. Proteins were separated by molecular weight on Bolt™4–12% Bis-Tris Plus precast gels in 1X Bolt™ morpholinepropanesulfonic acid (MOPS) running buffer for 30 min at 60 V, followed by 90 min at 80V in the presence of Chameleon™Duo Pre-stained Protein ladder. Membranes were blocked in 5% (w/v) instant nonfat dry milk in 1X tris-buffered saline (TBS) for 1 h at room temperature under gentle agitation. Gels were transferred onto membranes using a iBlot® 2 device (Thermo Fisher Scientific, Pleasanton, CA) set at 20V for 7 min. Membranes were subsequently washed 4 times for 3 min in 1X TBS supplemented with 0.05% Tween 20 (TBST) and incubated overnight at 4°C with primary antibodies resuspended in 5% (w/v) milk in TBS. Primary antibodies dilutions were optimized: β-actin (1:1000), β2m (1:750), LMP2 (1:500), LMP7 (1:250), MHC class I (1:250), PD-L1 (1:400), Jak1 (1:250), Jak2 (1:250), TAP1 (1:500). Membranes were once again washed 4 times for 3 min in TBST, and incubated with 1:2000 fluorescently labeled LI-COR secondary antibodies for 1 h at room temp. Membranes were washed 4 times for 3 min in TBST and imaged using an Odyssey infrared imaging system (version 3.0, LI-COR, Lincoln, NE).

### Whole exome sequencing (WES)

Genomic DNA was isolated from wt EMT6 and CRISPR/Cas9 KO cell lines as well as tails from Balb/c mice using a DNeasy Blood and Tissue Kit (Qiagen, Germantown, MD) following the manufacturer’s protocol. The sequencing facility at Frederick National Laboratory for Cancer Reseach performed the WES. Samples were pooled and sequenced on NovaSeq 6000 S1 run using Agilent SureSelect XT Mouse All Exon (Willmington, DE) and paired-end sequencing mode. A band-pass filter was set for reads between 163M and 205M and the Q30 was above 92%. The Dragen Bio-IT platform (Illumina, San Diego, CA) was used to map samples against the reference genome mm10 and to call variants [55]. MarkDuplicate calculated the library complexity (i.e., percentage of non-duplicate reads) by measuring the percentage of unique fragments in the mapped reads. Uniquely mapped reads were above 51% while duplicate reads were between 18% and 47%. Coverage statistics were measured using Dragen; more than 99% of the target region had a coverage above 20x. Raw sequencing depth coverage over the target region was between 501x and 628x, while the mapped sequencing depth coverage over target was between 267x to 327x. The mean insert size for these samples was calculated between 204 and 255 bases.

### WES somatic variant analysis

Somatic variant calling was performed using the Dragen Bio-IT platform set to paired mode. Variant calls were annotated and converted to a tabular format by the *vcf2maf* tool from the Memorial Sloan Kettering Cancer Center: https://github.com/mskcc/vcf2maf. The resulting data was filtered, and then visualized using the ‘maftools’ package (version 2.8.05) [56] and custom R scripts (version 4.1.0). Variants were filtered based on the following criteria: (a) depth in tumor cells > = 20; (b) variant allele frequency (VAF) in tumor cells > = 0.05; (c) variant allele depth in tumor cells > = 5; and (d) variant allele depth in normal < 5. Oncoplots were visualized using the R packages ‘ComplexHeatmap’ (version 2.8.0) [57]. Transcript and protein changes were named using the Human Genome Variation Society (HGVS) nomenclature [58] as reported by VEP. More information about this format can be found here: https://varnomen.hgvs.org/.

### Murine tumor studies

EMT6 (2.5 × 105) were implanted subcutaneously (s.c.) into the fourth inguinal mammary fat pad of Balb/c female mice. Starting on day 7 post-tumor implantation, tumor volumes were measured twice weekly using digital calipers and calculated as (length2 × width)/2. Throughout the studies, the overall animal health was monitored by animal facility veterinary staff and quantified using animal body weight.

### Necrosis quantification

Wt EMT6 tumors and their subsequent CRISPR/Cas9 knock out clones were harvested when the wt tumor volume reached 1000 mm^3^. Tumors were preserved in 10% formalin until further processing. Tumor sections were stained with haematoxylin and eosin (H&E) using standard procedures (HistoServ, Germantown, MD). Tumor sections were imaged with an AxioScan Z1 Slide Scanner (Zeiss, Dublin, CA) and necrosis was quantified using the QuPath v0.2.3 software by quantifying the areas of necrosis versus total tumor area.

### Flow cytometric analysis

Prior to staining, wt EMT6 cells and their subsequent CRISPR/Cas9 knockout clones were seeded (500,000 cells/well) onto 6-well plates and incubated for 24 h prior to treatment with 1 μg/mL of IFNγ for another 24 h period. Negative treatment controls were included in these studies. Cells were then washed two times with PBS, trypsinized (0.25% Trypsin-EDTA), and seeded onto 96-well plates (u-bottom) at a final density of 1 × 10^6^ cells per well. Similarly, wt EMT6 tumors and their subsequent CRISPR/Cas9 knockout clones were harvested when the wt tumor volume reached 1000 mm^3^. Tumor volume and weight were recorded at the time of harvest. Tumors were placed into gentleMACS® C-tubes (Miltenyi Biotec, Waltham, MA) containing 5 mL of RPMI-1640 medium supplemented with 5 mg/mL collagenase I (Worthington Biochemical, Lakewood, NJ), and 80 U/mL DNase (EMD Millipore, Burlington, MA). Tumors were cut into 1mm fragments prior to mechanical dissociation using a GentleMACS™Octo Dissociator (Miltenyi Biotec, Auburn, CA). The resulting mixture was incubated in a shaker at 37°C for 30 min. Tumors were once again subjected to mechanical dissociation, and then passed through a 70 μm cell strainer as a single-cell suspension. Red cell lysis was performed by treating the single-cell suspensions with 1 mL of ammonium-chloride-potassium (ACK) buffer for 1 min on ice.

Staining of cancer and immune cells (~1 × 10^6^) for flow cytometry was performed in the dark using the BD Cytofix/Cytoperm Kit (BD Biosciences, San Jose, CA) according to the manufacturer’s recommendations. Unless stated otherwise, all staining antibodies were diluted into BD Horizon brilliant stain buffer, including matched isotypes obtained from the manufacturers. Cells were first stained with 100 μL of LIVE/DEAD blue fluorescent reactive dye (Thermo Fisher Scientific, Pleasanton, CA) for 30 min at room temperature in PBS to differentiate between live and dead cells. After incubation, cells were washed with 100 μL of PBS, spun down at 400xg for 3 min, and the supernatant was removed by gently flicking the plate over a sink. To prevent background staining, cells were then blocked with 50 μL of anti-mouse CD16/CD32 in FACS buffer (PBS supplemented with 1% (v/v) heat inactivated FBS and 0.1% (w/v) sodium azide) for 15 min on ice. After incubation, cells were washed with 150 μL of FACS buffer, spun down at 400xg for 3 min, and the supernatant was removed by gently flicking the plate over a sink. Fifty μL of antibody cocktails were added to the cells for 30 min on ice. If intracellular staining was required, cells were incubated with 100 μL of BD cytofix/cytoperm for 30 min on ice, washed twice with 1X BD Perm/Wash, and incubated for 30 min on ice with the intracellular staining antibodies resuspended in BD Perm/Wash. Cells were washed as previously stated with FACS buffer prior to storage in 200 μL FACS buffer at 4°C until their acquisition on a LSRII Fortessa flow cytometer (BD Biosciences, San Jose, CA) with FACS Diva v 9.0 software. Results were analyzed with FlowJo Analysis Software v9.9.6 (Becton, Dickinson & Company, Ashland, OR). Cell populations were identified using gating strategies shown in S2 Fig in S1 File. All frequencies of phenotypic proteins were generated relative to the frequency of respective isotype, typically set between 1 and 5%.

### Quantification and statistical analysis

Statistical analyses were performed using GraphPad Prism (v8; GraphPad Software; San Diego, CA). All data points represent the mean ± SEM and p ≤ 0.05 was considered significant. Statistical differences between two sets of data were quantified through a two-tailed Student’s t-test. One-way ANOVA with Tukey’s post hoc test was performed to determine statistical differences among dataset containing more than two groups. Analysis of tumor growth curves was conducted using two-way analysis of variance (ANOVA). Statistical p values are denoted in the figures as follow: * p ≤ 0.05; ** p ≤ 0.01; *** p ≤ 0.001, **** p ≤ 0.0001. All statistical details (i.e., number of biological and experimental replicates) of experiments are described in the figure legends.

## Supporting information

S1 File Supplemental file. Supplemental Figures, Tables, and list of resources.
(PDF)

S2 File S1 raw images. Annotated western blots.
(PDF)
