# Supplementary material for: Generation of murine tumor models refractory to αPD-1/-L1 therapies due to defects in antigen processing/presentation or IFNγ signaling using CRISPR/Cas9
Source: PLoS One. 2024 Mar 1;19(3):e0287733. doi: 10.1371/journal.pone.0287733 (PMC10906908; doi:10.1371/journal.pone.0287733)
Supplement: S1 File — Supplemental Figures, Tables, and list of resources. (PDF) [file pone.0287733.s001.pdf]

Supplementary Figures and Tables

“Generation of murine tumor models refractory to αPD-1/-L1 therapies due to defects in antigen processing/presentation or IFNγ signaling using CRISPR/Cas9”

Paul L. Chariou, Christine M. Minnar, Mayank Tandon, Mary R. Guest, Raj Chari, Jeffrey Schlom, Sofia R. Gameiro

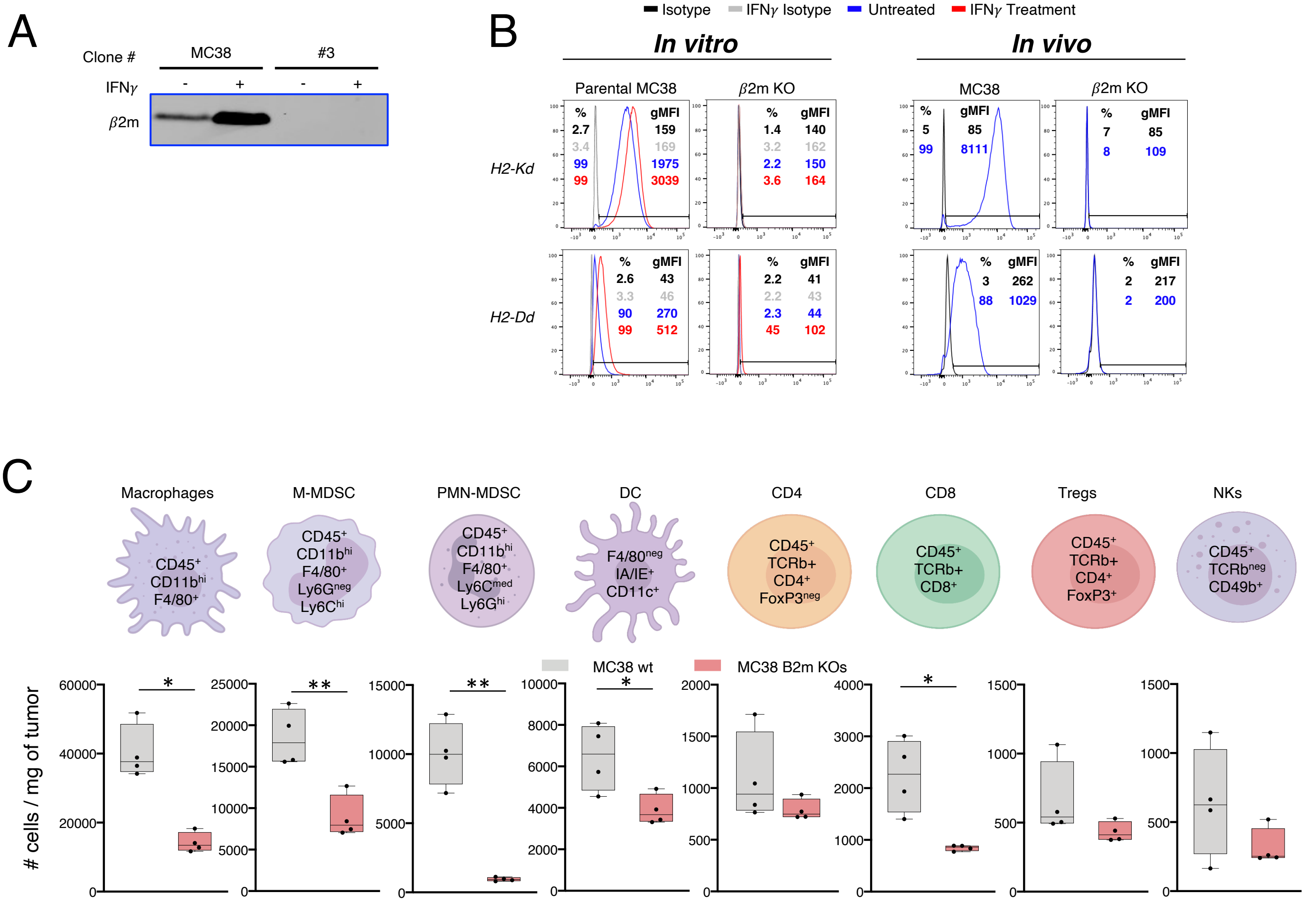

S1 Fig: A) Western blot quantification of β2m protein expression in the parental MC38 and β2m KO clone 3 previously incubated for 24 hours in the presence or absence of murine IFNγ. **B**) Representative histograms of cell surface expression of MHC class I haplotypes (H2-Kd and H2-Dd) *in vitro* and *in vivo* using untreated parental MC38 and the β2m KO clone compared to isotype controls. **C**) Flow cytometric analysis of the number of macrophages, mononuclear myeloid derived suppressor cells (M-MDSC), polymorphonuclear myeloid derived suppressor cells (PMN-MDSC), Dendritic cells (DC), $CD4^+$ T-lymphocytes cells (CD4), regulatory CD4 T-lymphocytes (Tregs) , $CD8^+$ T-lymphocytes cells (CD8), and natural killer cells (NK) per milligram of tumor. Tumors were extracted when their volume reached approximately 1000 mm^3. Statistics are two-tailed T-test. * = $p < 0.05$, ** = $p < 0.01$, *** = $p < 0.001$, **** = $p < 0.0001$.

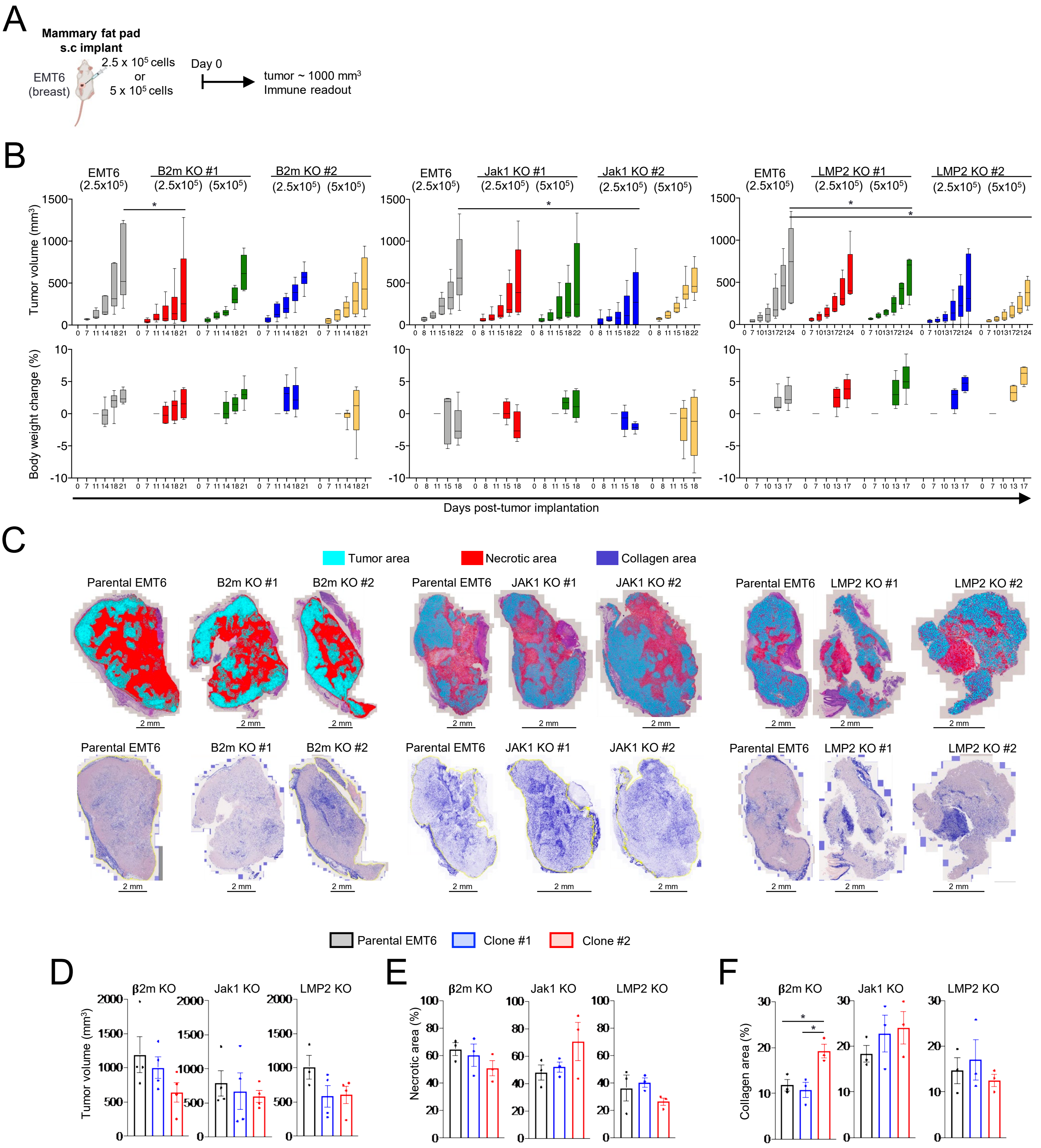

S2 Fig. Tumor growth in syngeneic Balb/C mice. **(A)** Generic treatment schedule for all tumor models. Mice were euthanized for analysis when tumors reached ~1000 mm^3. **(B)** Box and whisker plots corresponding to the tumor growth (top) and body weight (bottom) of parental EMT6 (**n=6**), b2m KO (**n=6**), Jak1 KO (**n=6**), and LMP2 KO (**n=6**) tumor-bearing mice treated as depicted in the schematic in Figure 3A. **(C)** Representative hematoxylin and eosin (H&E; top) and trichrome (bottom) images of parental and knocked out EMT6 tumors. Area of necrosis are indicated in red (as opposed to light blue), while areas of collagen are pseudo-colored in dark blue. Scale bar: 2 mm **(D)** Corresponding tumor volumes used at the time of **(E)** Necrosis quantification (**n = 4**) and **(F)** collagen quantification (**n = 4**). The growth curve and subsequent quantifications of the three KO models were performed once independently of each other. Statistics are two-way ANOVA (**B**) or ordinary one-way ANOVA with Tukey's multiple comparisons test (**D–F**). * = p < 0.05, ** = p < 0.01, *** = p < 0.001, **** = p < 0.0001. KO, knockout.

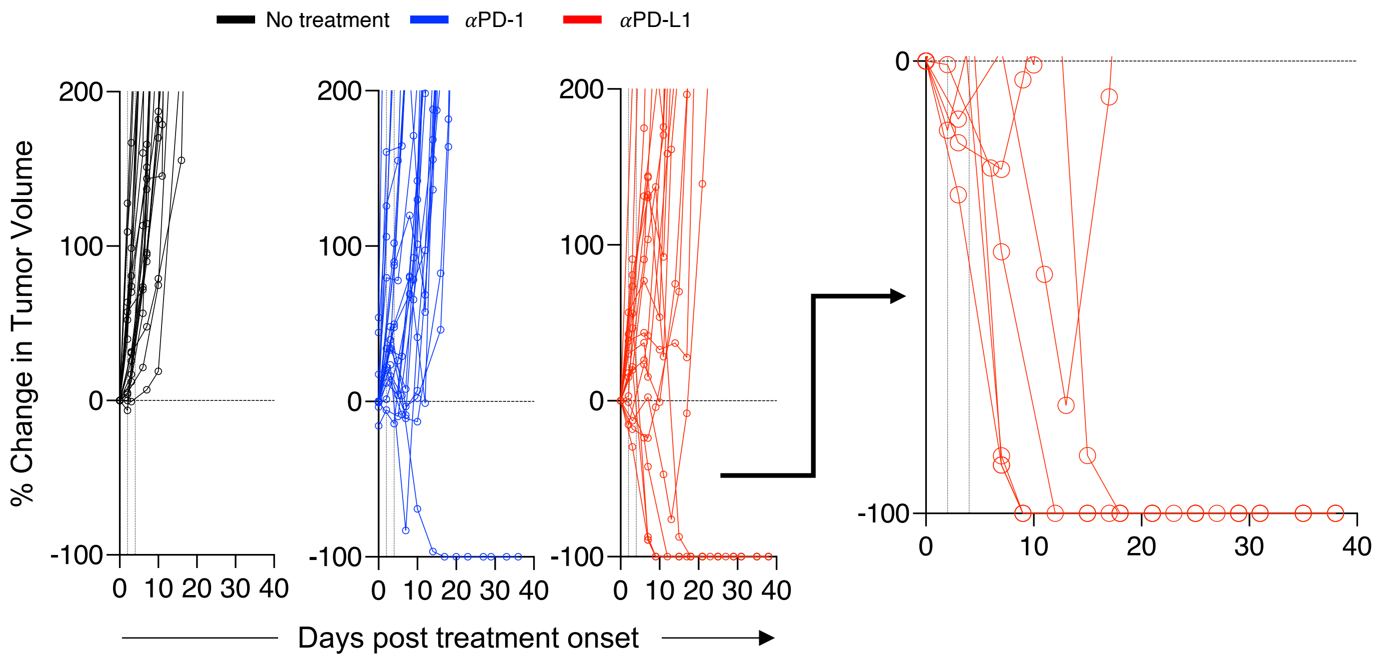

S3 Fig. Spider plots of individual EMT6 wt tumor growth in mice pertaining to survival data in Fig 5C. Graphs show % change in tumor volume in individual mice normalized to corresponding tumor size at the day of first treatment (day 0). Vertical black dashed lines depict days of αPD-1 or αPD-L1 dosing after the first treatment. Magnified graph on the right denotes % tumor volume change in mice treated with anti-PD-L1, where 5/19 mice attained prolonged cures.

Cell Type	Gating Strategy
CD4$^+$ T cells	Live>Singlets>LIVE/Dead Dye^{neg}>CD45$^+$>TCRb+>CD4$^+$>FoxP3^{neg}
Tregs	Live>Singlets>LIVE/Dead Dye^{neg}>CD45$^+$>TCRb$^+$>CD4$^+$>FoxP3$^+$
CD8$^+$ T Cells	Live>Singlets>LIVE/Dead Dye^{neg}>CD45$^+$>TCRb$^+$>CD8$^+$
DC	Live>Singlets>LIVE/Dead Dye^{neg}>CD45$^+$>F4/80^{neg}>IA/IE$^+$>CD11c$^+$
Macrophages	Live>Singlets>LIVE/Dead Dye^{neg}>CD45$^+$>CD11b^{hi}>F4/80$^+$
PMN-MDSC/Neutrophils	Live>Singlets>LIVE/Dead Dye^{neg}>CD45$^+$>CD11b^{hi}>F4/80$^+$>Ly6C^{med}>Ly6G^{hi}
NK	Live>Singlets>LIVE/Dead Dye^{neg}>CD45$^+$>TCRb^{neg}>CD49b$^+$
M-MDSC	Live>Singlets>LIVE/Dead Dye^{neg}>CD45$^+$>CD11b^{hi}>F4/80$^+$>Ly6G^{neg}>Ly6C^{hi}
Non-immune/Tumor cells	Live>Singlets>LIVE/Dead Dye^{neg}>CD45^{neg}

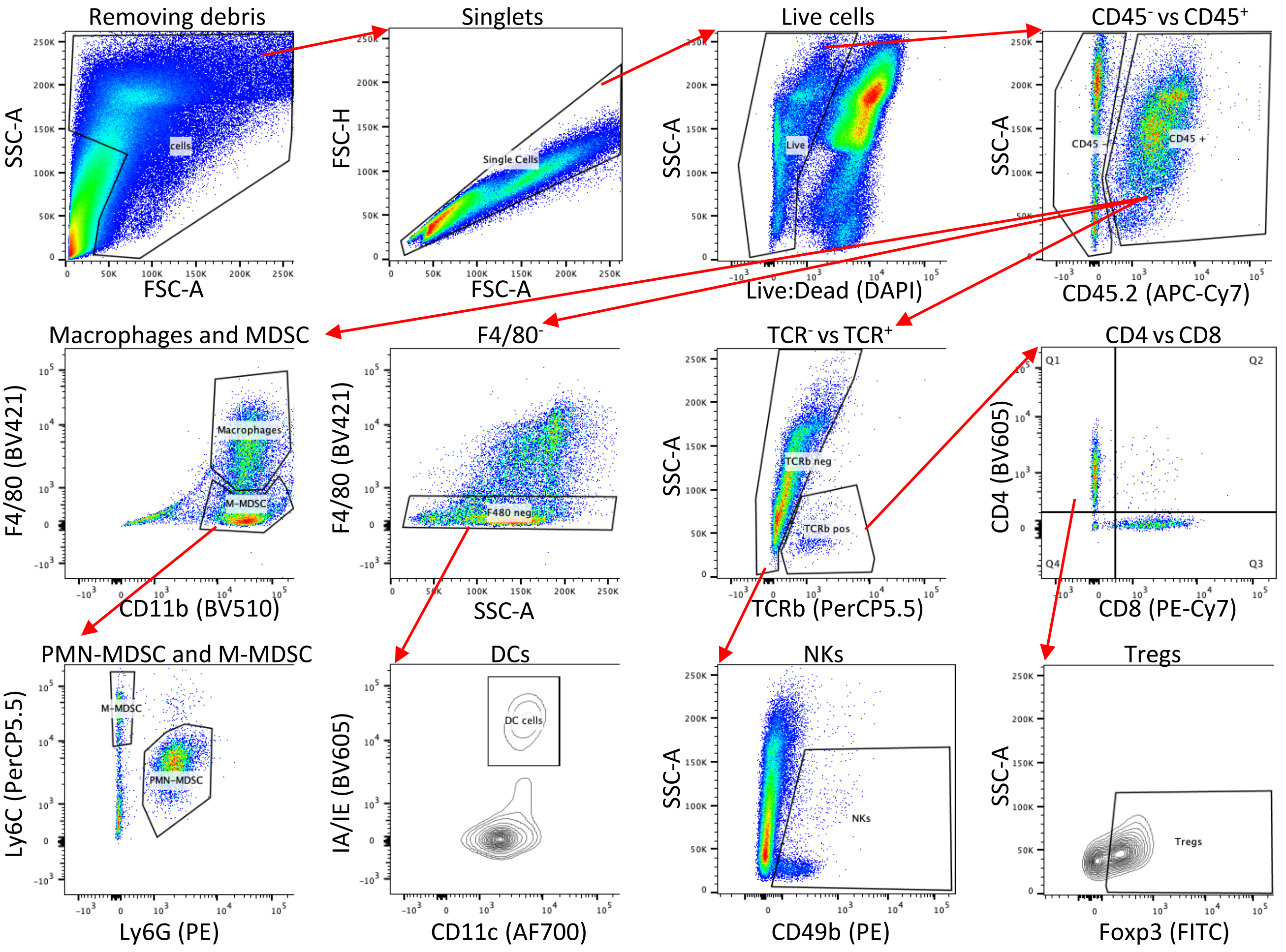

S4 Fig. Flow cytometry gating strategy.

DC, dendritic cells; M-MDSC, monocytic myeloid-derived suppressor cells; NK, natural killer cells; PMN-MDSC, polymorphonuclear myeloid-derived suppressor cells; Tregs, regulatory T cells.

Gene	Candidate	Target site (PAM in bold)
B2m	1756	AGTATACTCACGCCACCCAC**CGG**
B2m	1757	TCACGCCACCCACCGGAGAA**TGG**
B2m	1758	CACGCCACCCACCGGAGAAT**GGG**
B2m	1759	TCGGCTTCCCATTCTCCGGT**GGG**
B2m	1760	GGTCTTGGGCTCGGCCATAC**TGG**
B2m	1761	CCGAGCCCAAGACCGTCTAC**TGG**
Jak1	1780	ATGACAACGAACAGTCTGTA**TGG**
Jak1	1781	TGGATGACAAAACGTCTCTC**CGG**
Jak1	1782	TCCGAACCGAATCATCACTG**TGG**
Jak1	1783	ACGATGAGAGCACCAAGCTC**TGG**
Jak1	1784	CTTGGTGCTCTCATCGTACA**GGG**
Jak1	1785	TCTCATCGTACAGGGCGAAG**AGG**
Psmb9	1809	GGAGTTTGACGGGGGTGTCG**TGG**
Psmb9	1810	ATGGCAGTGGAGTTTGACGG**GGG**
Psmb9	1811	TTCCGTCCGGAACGAGCCGG**CGG**
Psmb9	1812	TACCGCCGGCTCGTTCCGGA**CGG**
Psmb9	1813	GTCCGGAACGAGCCGGCGGT**AGG**
Psmb9	1814	CACCTACCGCCGGCTCGTTC**CGG**

S1 Table. Candidate guide RNAs targeting *B2m*, *Jak1*, and *Psmb9* were identified using the sgRNA Scorer 2.0 algorithm. Candidates highlighted in grey were used in further experiments.
PAM, protospacer adjacent motif.

S2 Table. Supplementary Resources

REAGENT or RESOURCE	SOURCE	IDENTIFIER
**Antibodies**		
Rabbit monoclonal anti-mouse β2m (clone EP2978Y)	Abcam	Cat#Ab75853<br>Lot#GR3315403-6<br>RRID:AB_1523204
Rabbit monoclonal anti-mouse LMP2 (clone EPR22042)	Abcam	Cat#Ab242061<br>Lot#GR3373421-4<br>RRID:AB_2893429
Mouse monoclonal anti-mouse MHC class I H2-Dd (clone 34-1-2S)	Abcam	Cat#Ab174603<br>Lot#GR3419724-2<br>RRID:AB_2744693
Rabbit monoclonal anti-mouse PD-L1 (clone EPR20529)	Abcam	Cat#Ab213480<br>Lot#gr3217502-1<br>RRID:AB_2773715
Rabbit monoclonal anti-mouse Jak2 (clone EPR108(2))	Abcam	Cat#Ab108596<br>Lot#GR3253328-3<br>RRID:AB_10865183
Rat monoclonal anti-mouse CD4 (clone RM4-5)	BD Biosciences	Cat#563151<br>Lot#0314025<br>RRID:AB_2687549
Rat monoclonal anti-mouse CD11b (clone M1/70)	BD Biosciences	Cat#562950<br>Lot#0209403<br>RRID:AB_2737913
Mouse monoclonal anti-mouse H-2Dd (clone 34-2-12)	BD Biosciences	Cat#110606<br>Lot#B243258<br>RRID:AB_394938
Rat monoclonal anti-mouse IFNγRI (clone GR20)	BD Biosciences	Cat#740897<br>Lot#1060155<br>RRID:AB_2740545
Rat monoclonal anti-mouse Ly6G (clone 1A8)	BD Biosciences	Cat#551461<br>Lot#5362993<br>RRID:AB_394208
Rat monoclonal anti-mouse IA/IE (clone M5/114.15.2)	BD Biosciences	Cat#562363<br>Lot#1152629<br>RRID:AB_11153297
Rat monoclonal anti-mouse CD8α (clone 53-6.7)	BioLegend	Cat#100706<br>Lot#B195504<br>RRID:AB_312745
Armenian Hamster monoclonal anti-mouse CD11c (clone N418)	BioLegend	Cat#117320<br>Lot#B348039<br>RRID:AB_117320
Mouse monoclonal anti-mouse CD45.2 (clone 104)	BioLegend	Cat#109824<br>Lot#B294042<br>RRID:AB_830789
Rat monoclonal anti-mouse CD49b (clone DX5)	BioLegend	Cat#108908<br>Lot#B271646<br>RRID:AB_313415
Mouse monoclonal anti-mouse H-2Kd (clone SF1-1.1)	BioLegend	Cat#116623<br>Lot#B354650<br>RRID:AB_2565656

Rat monoclonal anti-mouse Ly6C (clone HK1.4)	BioLegend	Cat#128028<br>Lot#B282011<br>RRID:AB_10897805
Armenian Hamster monoclonal anti-mouse TCRβ (clone H57-597)	BioLegend	Cat#109228<br>Lot#B272829<br>RRID:AB_1575173
Rat monoclonal anti-mouse F4/80 (clone BM8)	BioLegend	Cat#123132<br>Lot#B265669<br>RRID:AB_11203717
Mouse monoclonal anti-mouse Jak1 (clone D1T6W)	Cell Signaling	Cat#50996S<br>Lot#50996S<br>RRID:AB_2716281
Rabbit monoclonal anti-mouse β-actin (clone D6A8)	Cell Signaling	Cat#8457S<br>Lot#8457L<br>RRID:AB_10950489
Rabbit monoclonal anti-mouse LMP7 (clone D1K7X)	Cell Signaling	Cat#13635S<br>Lot#13635S<br>RRID:AB_1523204
Rabbit polyclonal anti-mouse Tap1	Thermo Fisher Scientific	Cat#BS-2789R<br>Lot#BJ01222334<br>RRID:AB_10857457
Mouse monoclonal anti-mouse CD274/PD-L1 (clone MIH5)	Thermo Fisher Scientific	Cat#25-5982-82<br>Lot#4341651<br>RRID:2573509
Rat monoclonal anti-mouse FoxP3 (clone FJK-16s)	Thermo Fisher Scientific	Cat#45-5773-82<br>Lot#2290357<br>RRID:AB_914351
IRDye CW Goat anti-Rat IgG Secondary Antibody	LI-COR	Cat# 926-32219<br>Lot# D10914-08<br>RRID:AB_1850025
IRDye CW Goat anti-Rabbit IgG Secondary Antibody	LI-COR	Cat# 926-32211<br>Lot# D10831-15<br>RRID:AB_621843
IRDye CW Goat anti-Mouse IgG Secondary Antibody	LI-COR	Cat# 926-32210<br>Lot#D10825-15<br>RRID:AB_621842
**Bacterial and virus strains**		
NEB® 5-alpha competent *E. coli*	New England Biolabs	Cat#C2987H
Endura competent *E.coli*	Lucigen	Cat#60242-1
**Commercial assays and reagents**		
Collagenase I	Worthington Biochemical	Cat# LS004197
DNAse I	EMD MIllipore	Cat#260913
MycoAlert mycoplasma detection Kit	Lonza	Cat#G7571
Quick extract	Lucigen	Cat#QE09050
CellTiter-Glo® luminescent cell viability assay	Promega	Cat#G7572
DNeasy blood and tissue Kit	Qiagen	Cat#69504
Lipofectamine® 3000 transfection kit	Thermo Fisher Scientific	Cat#L3000-015
Pierce™ BCA protein assay kit	Thermo Fisher Scientific	Cat#23225
PureLink HiPure plasmid maxiprep kit	Thermo Fisher Scientific	Cat#K210007

Zero blunt TOPO PCR cloning kit	ThermoFisher Scientific	Cat#450245
Genejet miniprep kit	ThermoFisher Scientific	Cat#K0502
**Deposited data**		
Whole exome sequencing raw data	This study	https://dataview.ncbi.nlm.nih.gov/object/PRJNA870222?reviewer=7rvf5115qoca3b0asocfsn3vfc
Whole exome sequencing code	This study	https://github.com/CCBR/CCBR-1144
Sanger sequencing raw data	This study	https://github.com/CCBR/CCBR-1144
TCGA data analysis code	This study	https://github.com/CCBR/CCBR-1144
**Cell lines**		
EMT6	ATCC	Cat#CRL-2755 RRID:AB_CVCL_1923
EMT6 B2m KO	This study	N/A
EMT6 Jak1 KO	This study	N/A
EMT6 LMP2 KO	This study	N/A
P19	ATCC	Cat#CRL-1825 RRID:AB_CVCL_2153
**Organisms/strains**		
Mouse Balb/C	NCI Frederick Cancer Research Facility	RRID:IMSR_APB:4790
**Recombinant DNA**		
Lenti-CRISPRv2GFP	Addgene	Cat#82416 ; RRID:Addgene_82416
**Software and algorithms**		
Dragen Bio-IT	Illumina	https://support.illumina.com/sequencing/sequencing_software/dragen-bio-it-platform.html
FlowJo, Version 10.7.1	Ashland, OR: Becton, Dickinson and Company	https://www.flowjo.com/solutions/flowjo
Geneious Prime 2020.1.2	Dotmatics	https://www.geneious.com/download/
Prism, Version 8.4.2	GraphPad	https://www.graphpad.com/scientific-software/prism
QuPath, Version 0.2.3	Bankhead et al. (2017)	https://qupath.github.io
sgRNA Scorer 2.0 algorithm	Frederick National Laboratory for Cancer Research	https://sgrnascorer.cancer.gov/

TCGABiolinks R package (version 2.20.1)	Colaprico et al. (2016)	https://bioconductor.org/packages/release/bioc/html/TCGAbiolinks.html
**Instruments**		
AxioScan Z1 Slide Scanner	Zeiss	N/A
BD LSR Fortessa™	BD Biosciences	Cat#647177
GentleMACS™Octo Dissociator	Miltenyi Biotec	Cat#130-095-937
iBlot® 2 device	Thermo Fisher Scientific	Cat#IB21001
MA900 cell sorter	Sony	N/A
MiSeq Sequencer	Illumina	Cat#SY-410-1003
Multimode Plate Reader EnVision	PerkinElmer	Cat#2105-0010
NanoDrop One	Thermo Fisher Scientific	Cat#ND-ONE-W
Novice 6000 S1	Illumina	N/A
Odyssey infrared imaging system	LI-COR	N/A
