# Supplementary material for: Generation of murine tumor models refractory to αPD-1/-L1 therapies due to defects in antigen processing/presentation or IFNγ signaling using CRISPR/Cas9
Source: PLoS One. 2024 Mar 1;19(3):e0287733. doi: 10.1371/journal.pone.0287733 (PMC10906908; doi:10.1371/journal.pone.0287733)

FIGURE 1 WESTERN BLOTS

Images acquired using an Odyssey infrared imaging system (version 3.0, LI-COR, Lincoln, NE)

D

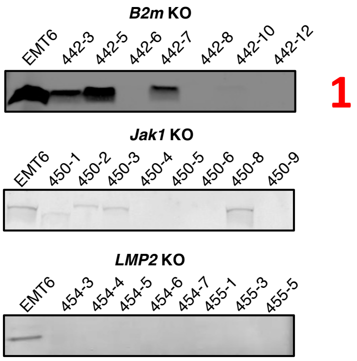

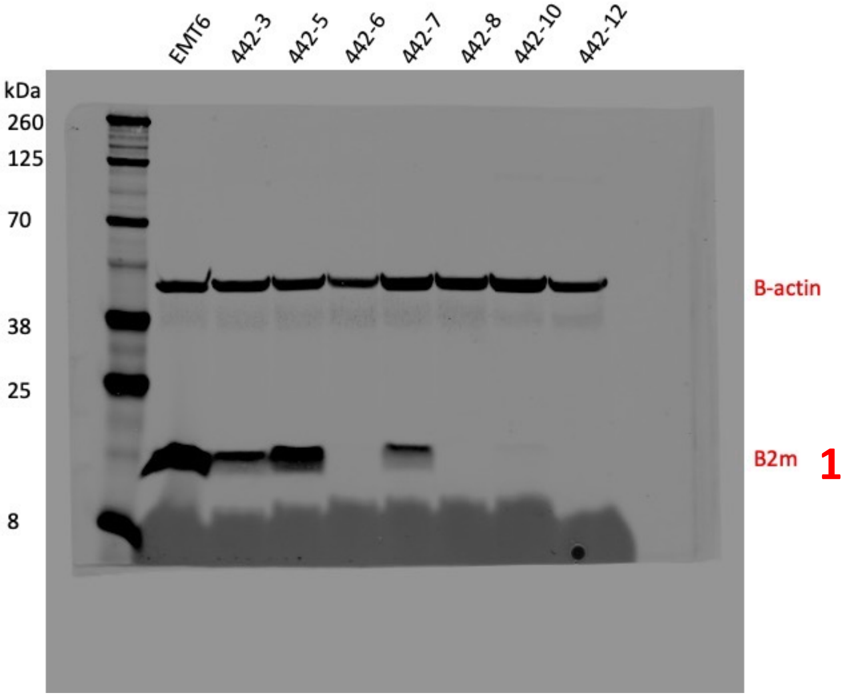

Same western blot acquired with higher exposure:

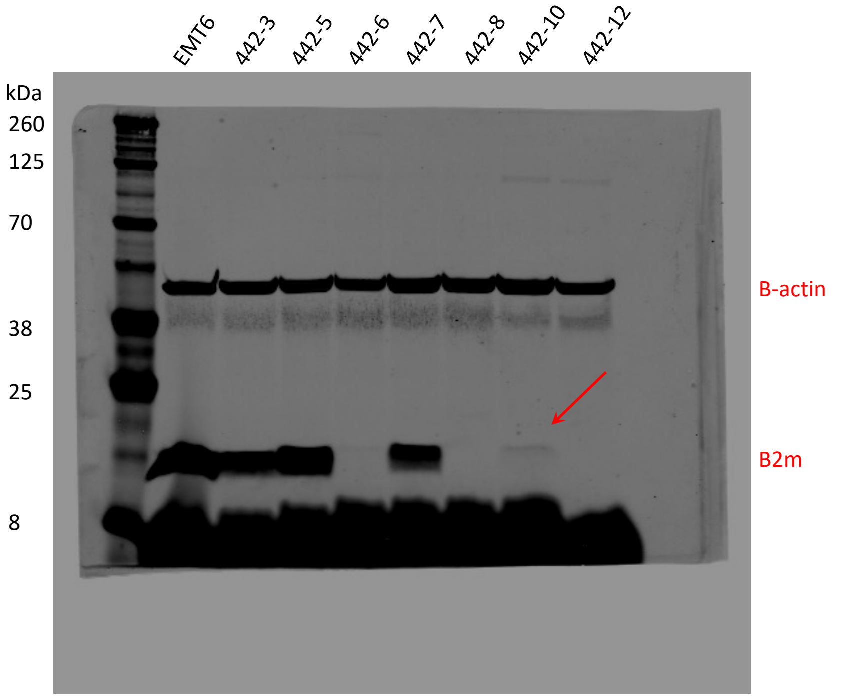

Note B2m positive expression in clone 442-10

FIGURE 1 WESTERN BLOTS

Images acquired using an Odyssey infrared imaging system (version 3.0, LI-COR, Lincoln, NE)

D

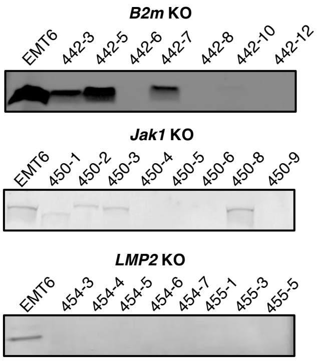

2

3

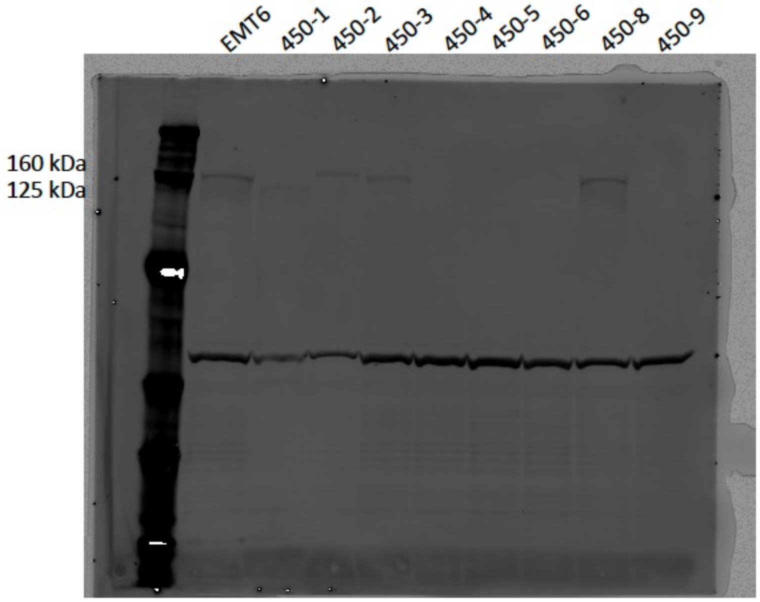

2

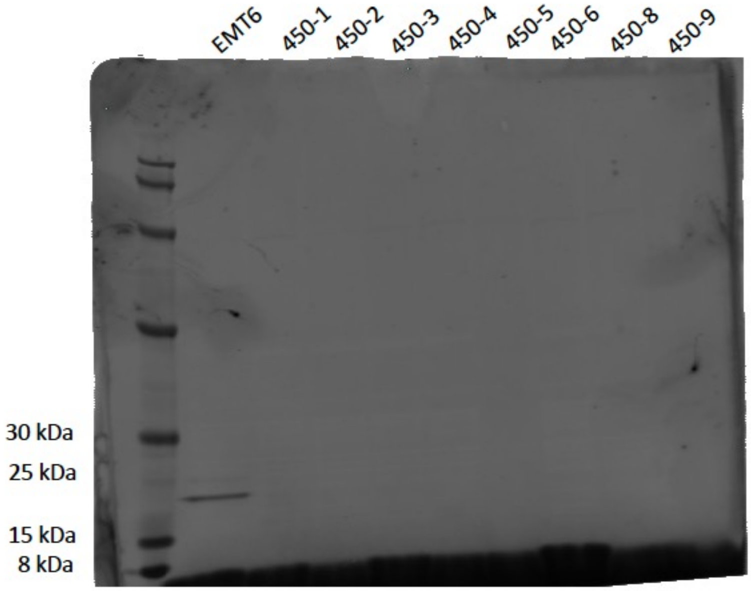

3

FIGURE 2 WESTERN BLOTS

Images acquired using an Odyssey infrared imaging system (version 3.0, LI-COR, Lincoln, NE)

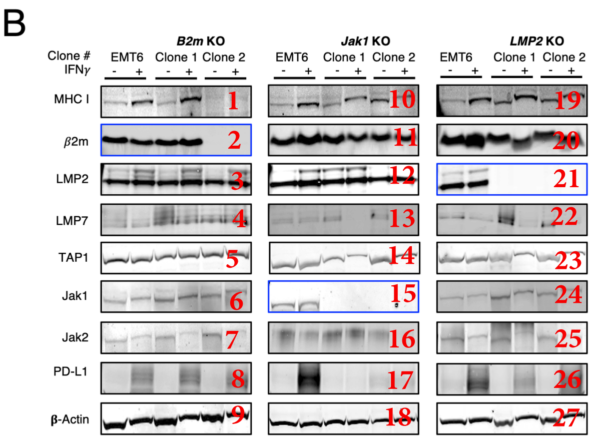

B2m KO: MHC I

EMT6 Clone 1 Clone 2

IFNγ + - + - + -

50 kDa

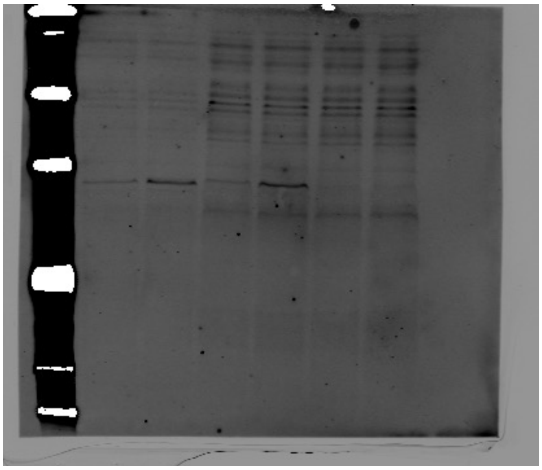

1

B2m KO: B2m

EMT6 Clone 1 Clone 2

IFNγ + - + - + -

50 kDa
38 kDa
30 kDa
25 kDa
15 kDa
8 kDa

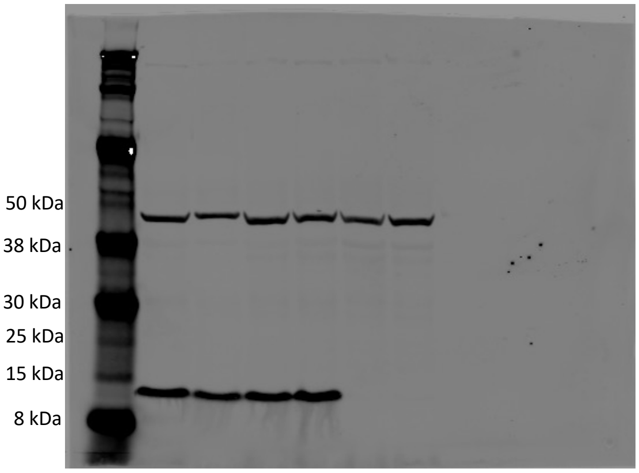

2

B2m KO: LMP2

EMT6 Clone 1 Clone 2

IFNγ + - + - + -

50 kDa
38 kDa
25 kDa
15 kDa

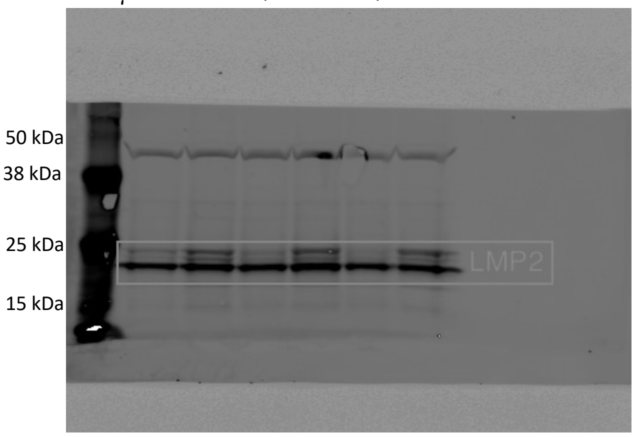

3

B2m KO: LMP7

EMT6 Clone 1 Clone 2

IFNγ + - + - + -

50 kDa
38 kDa
25 kDa
15 kDa

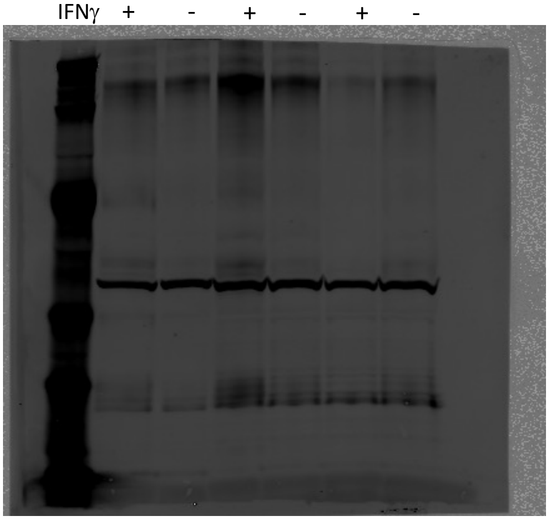

4

FIGURE 2 WESTERN BLOTS

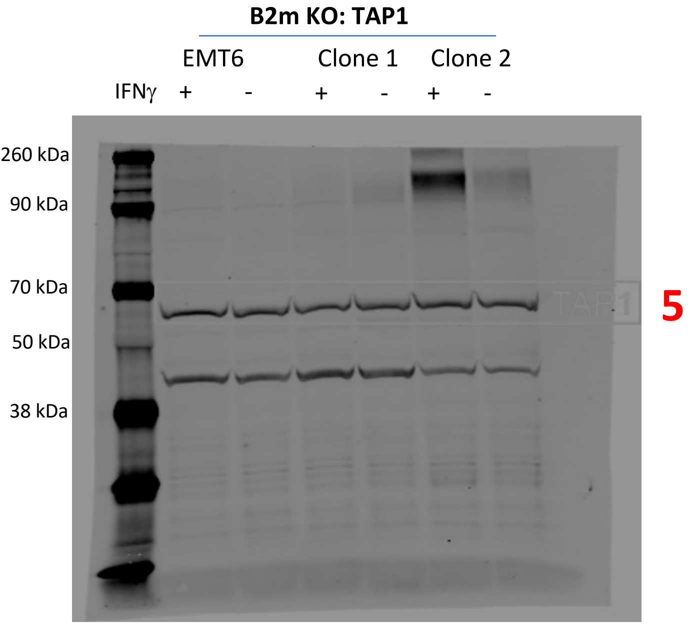

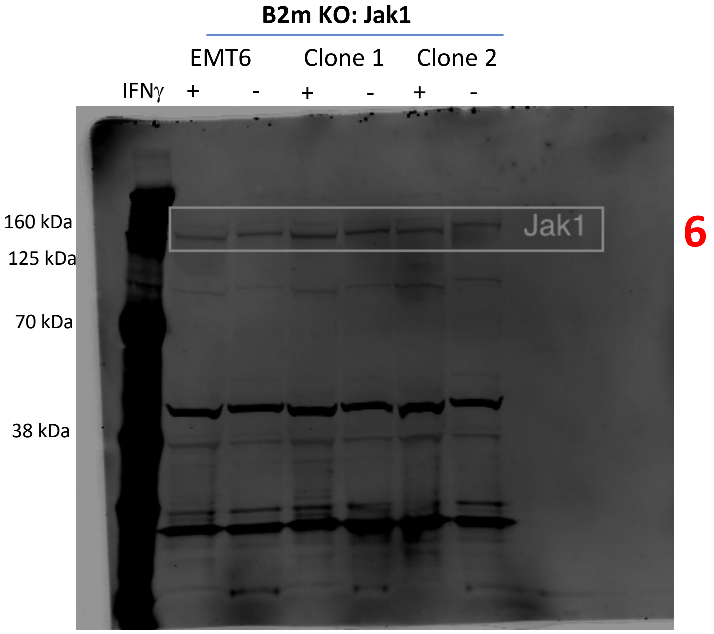

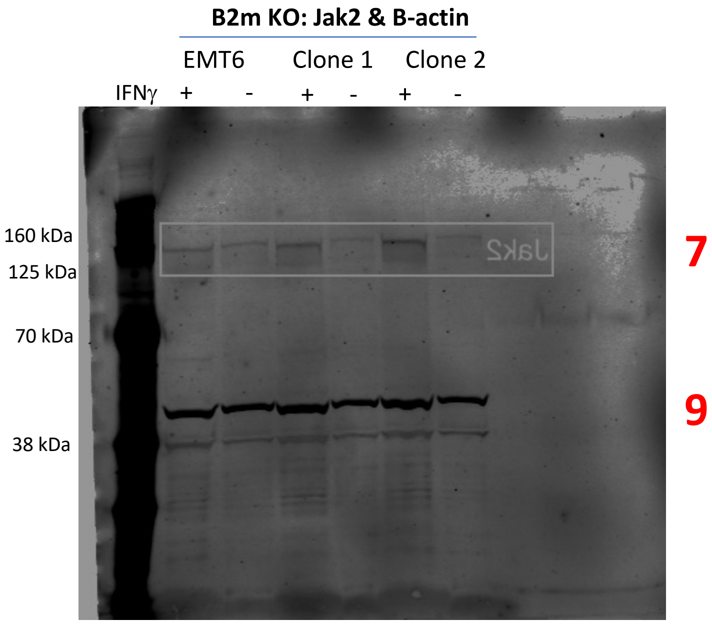

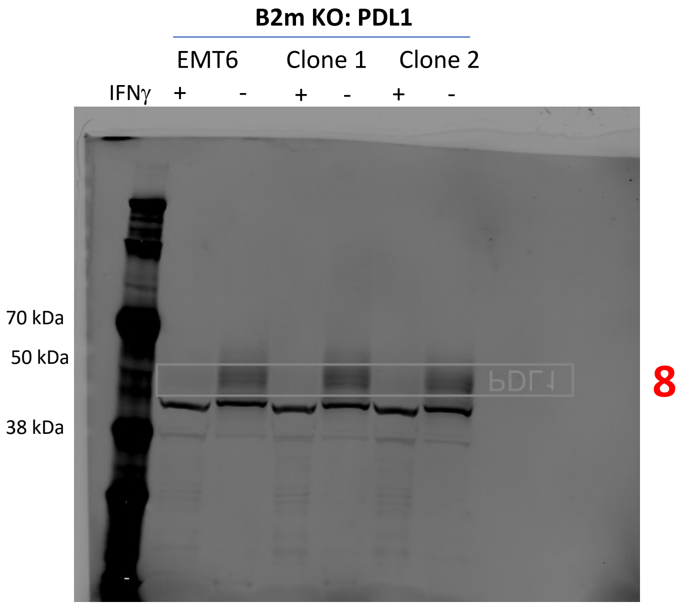

FIGURE 2 WESTERN BLOTS

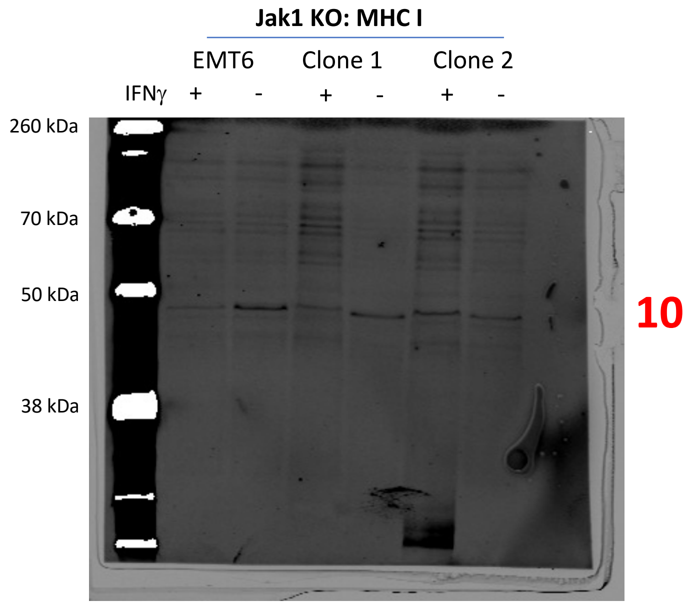

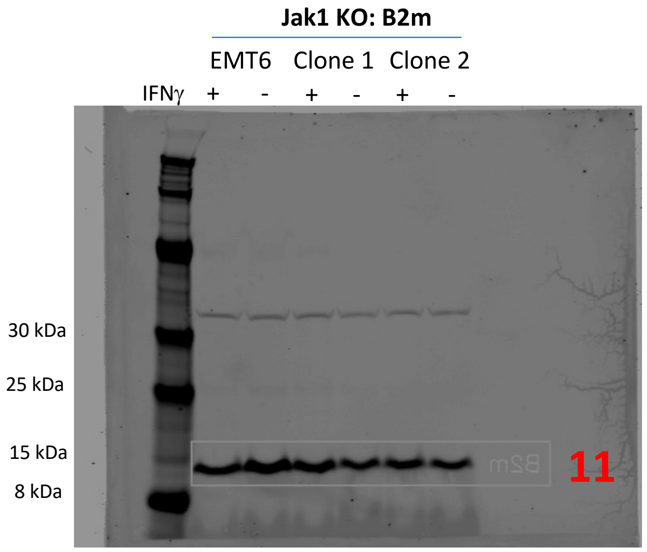

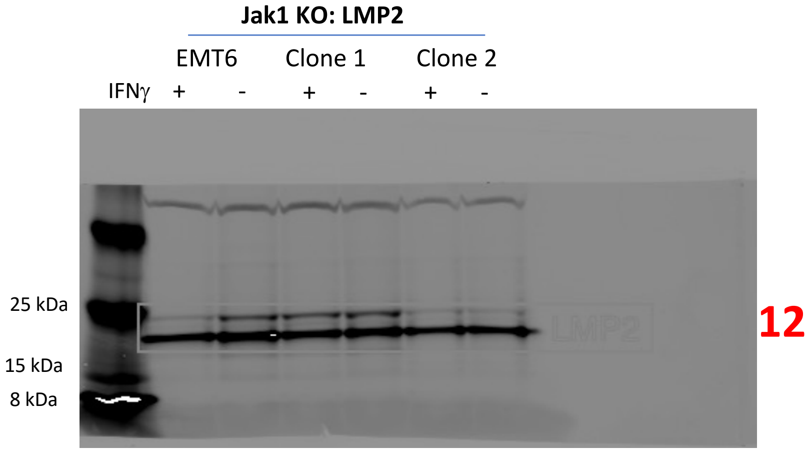

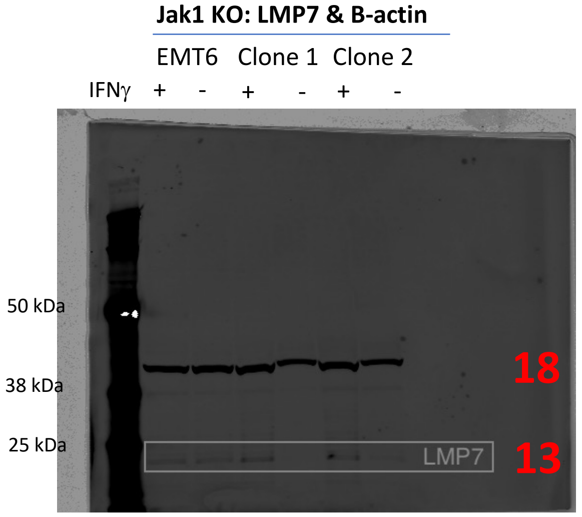

FIGURE 2 WESTERN BLOTS

Jak1 KO: TAP1

EMT6 Clone 1 Clone 2

IFNγ + - + - + -

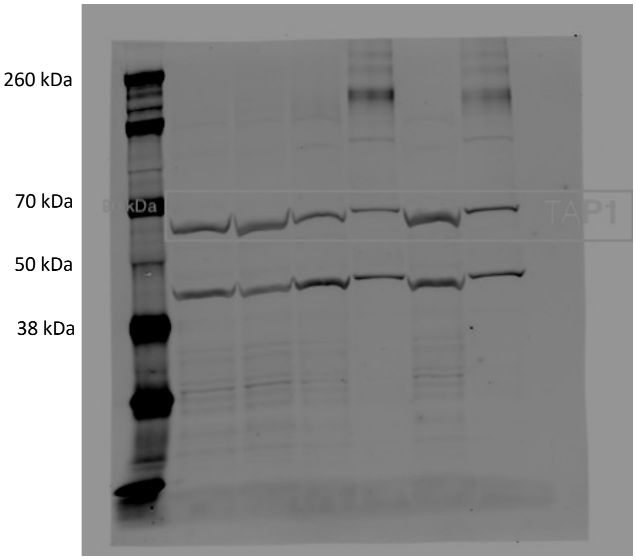

14

Jak1 KO: Jak1

EMT6 Clone 1 Clone 2 X X

IFNγ + - + - + -

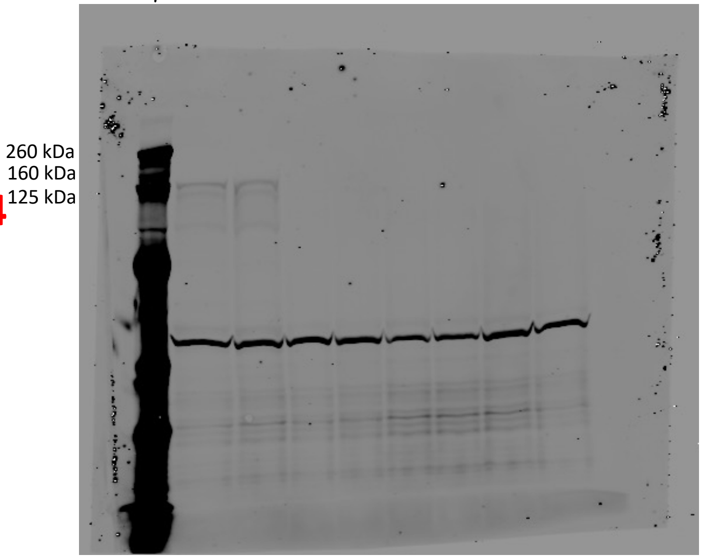

15

Jak1 KO: Jak2

EMT6 Clone 1 Clone 2

IFNγ + - + - + -

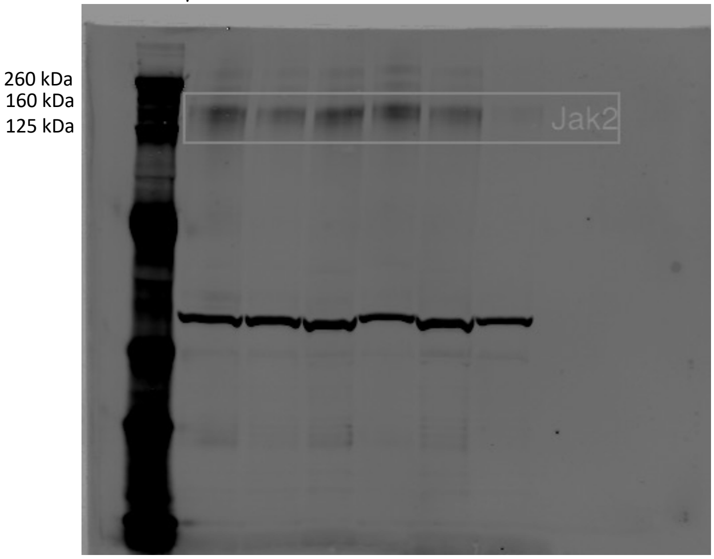

16

Jak1 KO: PDL1

EMT6 Clone 1 Clone 2

IFNγ + - + - + -

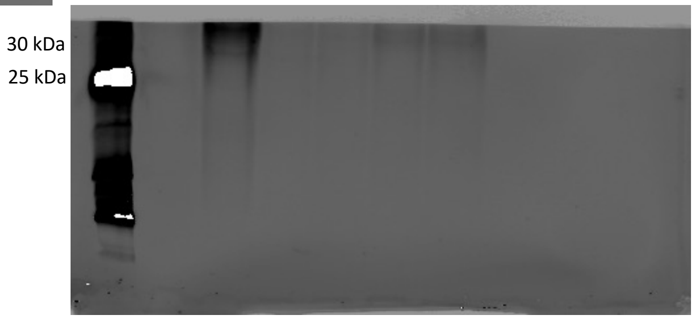

17

FIGURE 2 WESTERN BLOTS

LMP2 KO: MHC I

EMT6 Clone 1 Clone 2

IFNγ + - + - + -

50 kDa

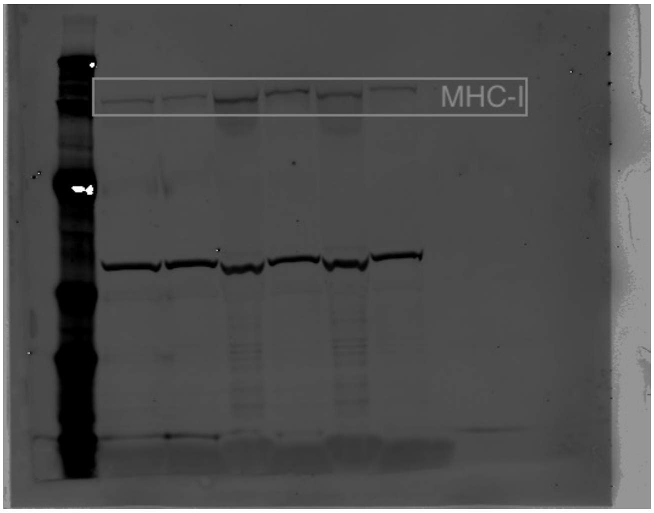

19

LMP2 KO: B2m

EMT6 Clone 1 Clone 2

IFNγ + - + - + -

50 kDa
38 kDa
30 kDa
25 kDa
15 kDa
8 kDa

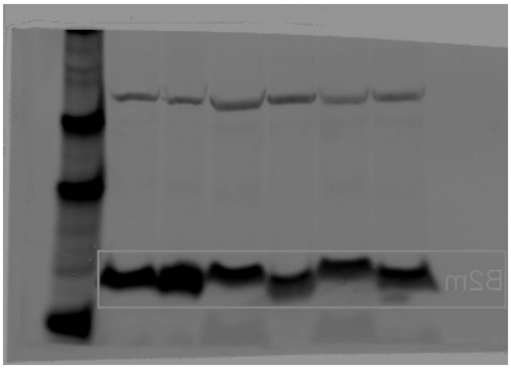

20

LMP2 KO: LMP2

EMT6 Clone 1 Clone 2

IFNγ + - + - + -

30 kDa
25 kDa
15 kDa

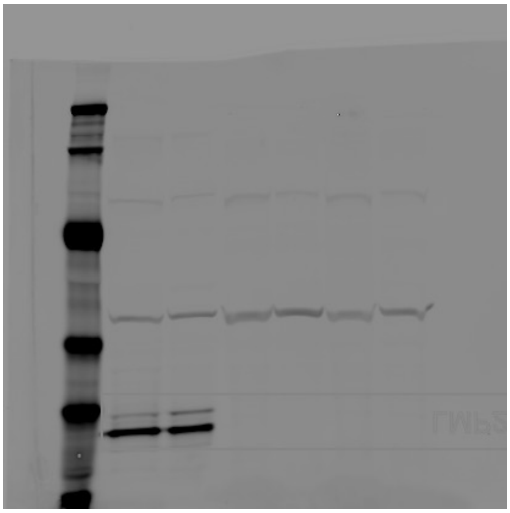

21

LMP2 KO: LMP7

EMT6 Clone 1 Clone 2

IFNγ + - + - + -

30 kDa
25 kDa
15 kDa

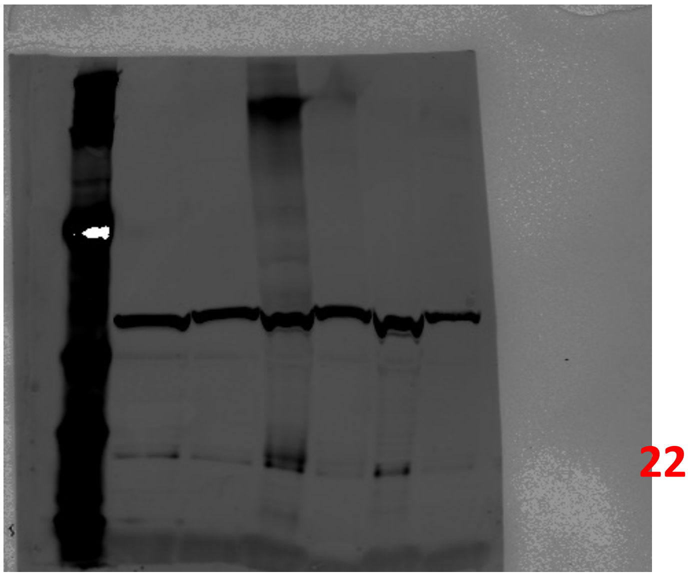

22

FIGURE 2 WESTERN BLOTS

LMP2 KO: TAP1

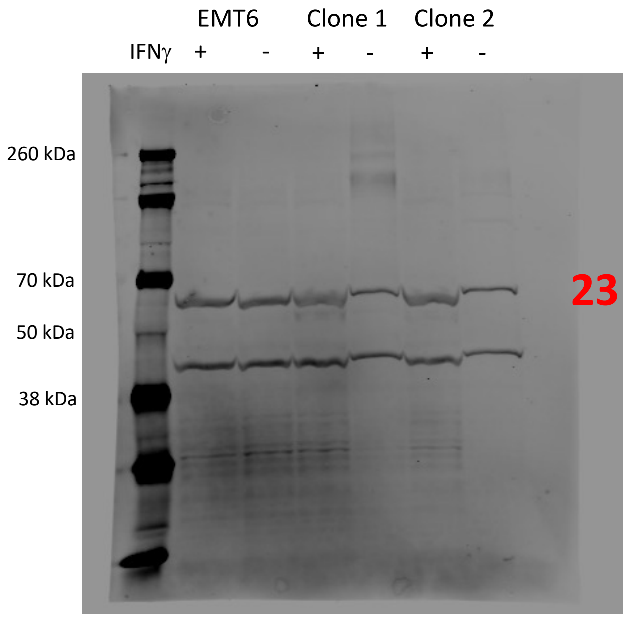

LMP2 KO: JAK1

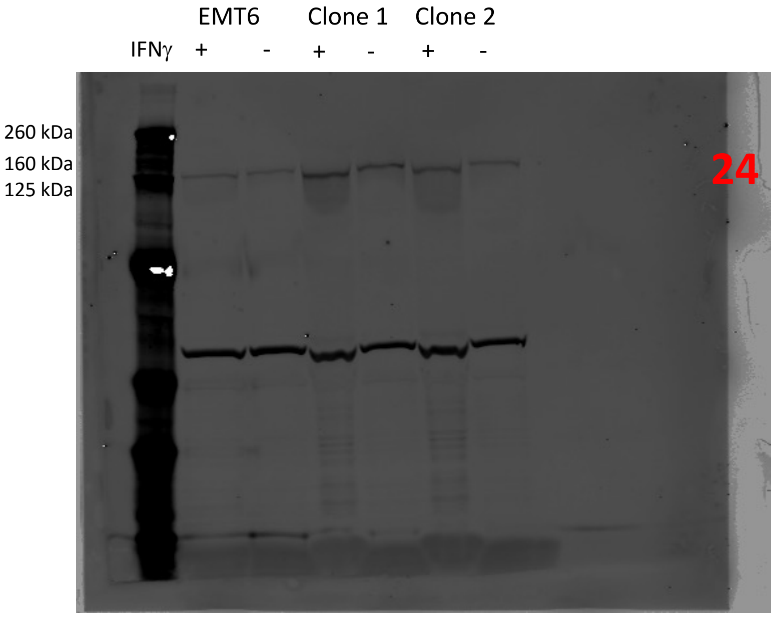

LMP2 KO: JAK2

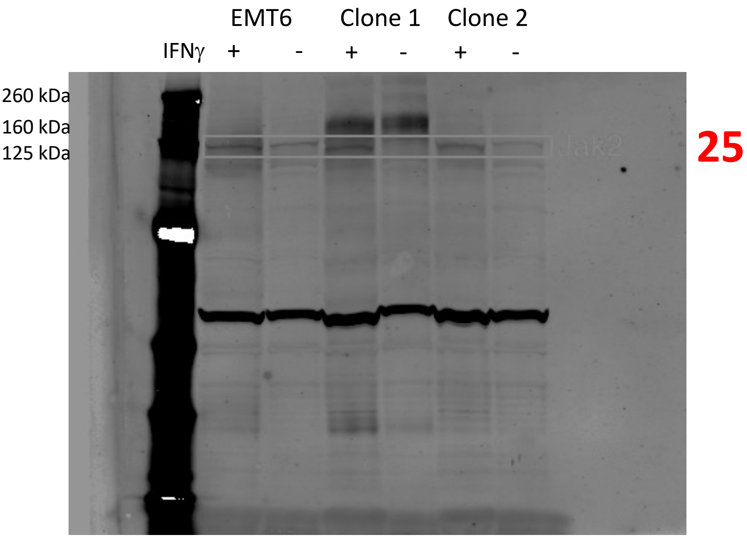

LMP2 KO: PDL1

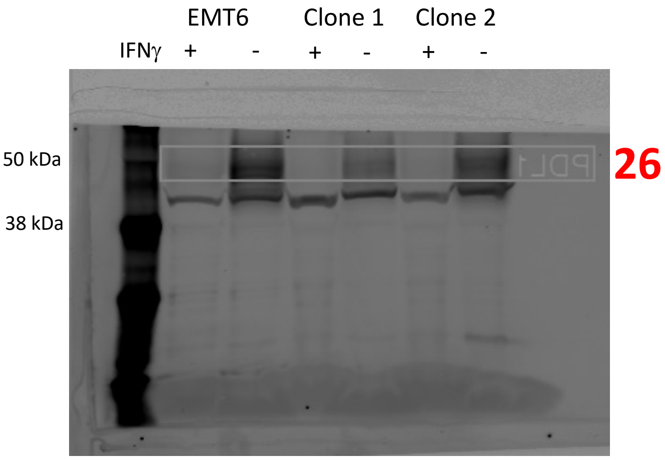

LMP2 KO: B-actin

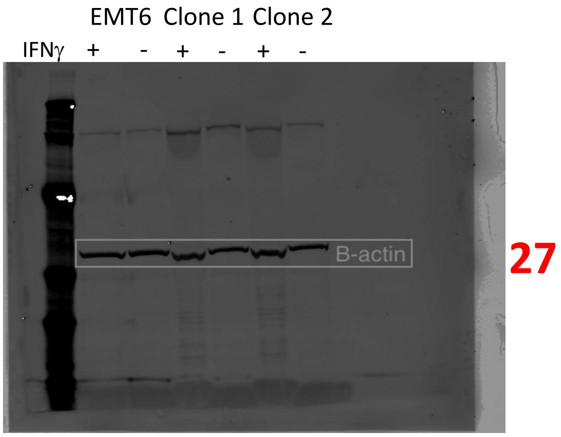

S1 FIG 1A WESTERN BLOT

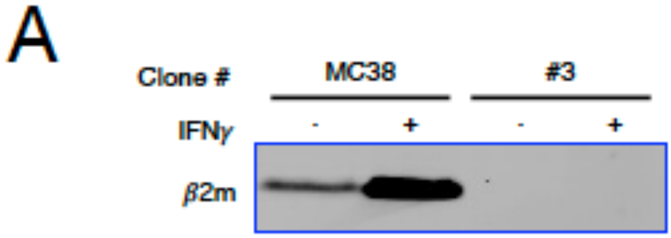

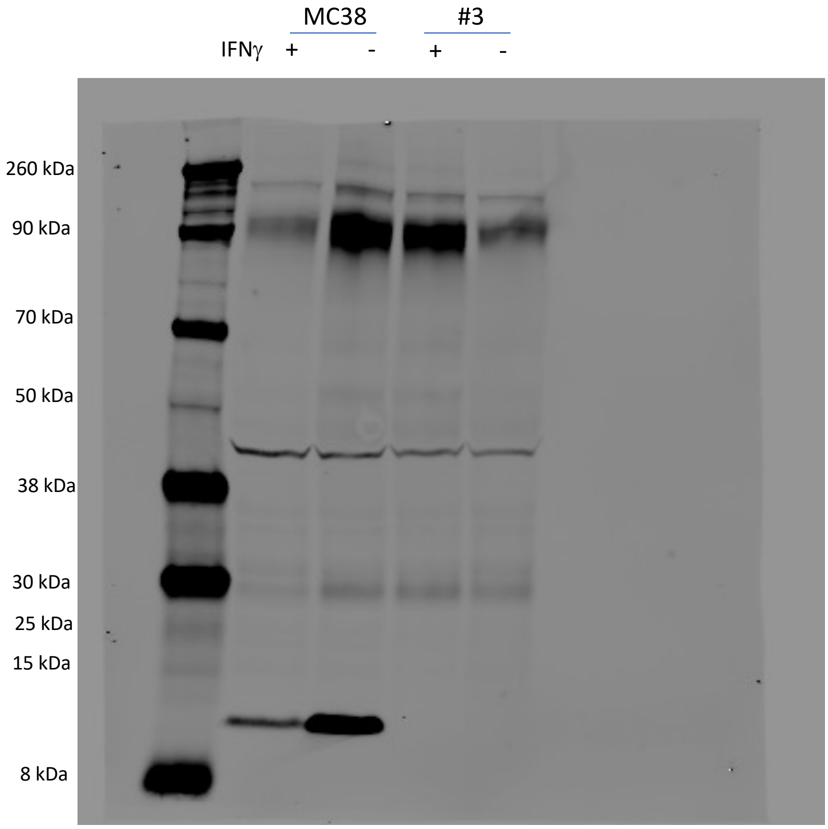

Supplement: S2 File — Annotated western blots. (PDF) [file pone.0287733.s002.pdf]
